# Renal clearable nanochelators for iron overload therapy

**DOI:** 10.1038/s41467-019-13143-z

**Published:** 2019-11-13

**Authors:** Homan Kang, Murui Han, Jie Xue, Yoonji Baek, JuOae Chang, Shuang Hu, HaYoung Nam, Min Joo Jo, Georges El Fakhri, Michael P. Hutchens, Hak Soo Choi, Jonghan Kim

**Affiliations:** 1 Gordon Center for Medical Imaging, Department of Radiology, Massachusetts General Hospital and Harvard Medical School, Boston, MA 02114 USA; 2 Department of Pharmaceutical Sciences, Northeastern University, Boston, MA 02115 USA; 3 Anesthesiology and Perioperative Medicine, Oregon Health & Science University, Portland, OR 97239 USA; 4 Portland Veterans Affairs Medical Center, Portland, OR 97239 USA

**Keywords:** Nanoparticles, Haematological diseases, Drug development, Biomedical materials

## Abstract

Iron chelators have been widely used to remove excess toxic iron from patients with secondary iron overload. However, small molecule-based iron chelators can cause adverse side effects such as infection, gastrointestinal bleeding, kidney failure, and liver fibrosis. Here we report renal clearable nanochelators for iron overload disorders. First, after a singledose intravenous injection, the nanochelator shows favorable pharmacokinetic properties, such as kidney-specific biodistribution and rapid renal excretion (>80% injected dose in 4 h), compared to native deferoxamine (DFO). Second, subcutaneous (SC) administration of nanochelators improves pharmacodynamics, as evidenced by a 7-fold increase in efficiency of urinary iron excretion compared to intravenous injection. Third, daily SC injections of the nanochelator for 5 days to iron overload mice and rats decrease iron levels in serum and liver. Furthermore, the nanochelator significantly reduces kidney damage caused by iron overload without demonstrating DFO’s own nephrotoxicity. This renal clearable nanochelator provides enhanced efficacy and safety.

## Abstract (web summary)

The build-up of iron in the body can have serious consequences; current treatment therapies suffer from adverse side effects and toxicity. Here, the authors developed renal clearable nanochelators with improved pharmacodynamics and demonstrated their efficacy and safety in iron overload animal models.

## Introduction

Iron is an essential metal nutrient, but excess iron increases oxidative stress and promotes tissue damage by catalysis of hydroxyl radicals to accelerate lipid peroxidation^1^. Consequently, it increases the risk of heart failure, liver cirrhosis and cancer, arthritis, dyslipidemia, diabetes, and gonadal dysfunction^2,3^, and may be associated with several neurodegenerative diseases (e.g. Alzheimer’s and Parkinson’s diseases)^4–7^. Tens of millions of people are affected by various types of iron overload disorders. Hereditary hemochromatosis (a.k.a. primary iron overload) is a genetic disorder characterized by increased absorption of iron into the intestines and deposition of iron into parenchymal tissues, resulting in premature death^2,3^. In addition, patients with hemoglobinopathies, including thalassemia major, sickle cell anemia, aplastic anemia, myelodysplastic syndrome, or Diamond–Blackfan anemia, require repeated blood transfusions, which can cause secondary iron overload due to the absence of the effective physiological pathway of iron excretion^8^.

The chelation therapy has been widely used to improve conditions mainly in transfusion-dependent patients with iron overload^9,10^. Chelating agents form iron complexes that promote iron excretion, clear plasma non-transferrin-bound iron, remove excess iron from cells, and restore iron levels in the body to safe levels. However, Food and Drug Administration (FDA)-approved iron chelators such as deferoxamine (DFO), deferiprone, and deferasirox have unfavorable pharmacokinetics (PK) and pharmacodynamics (PD), nonspecific tissue distribution, and significant adverse effects^11^ (Supplementary Table 1). For example, native DFO has a very short half-life (e.g. 5–15 min in rodents^12,13^) and distributes into non-target tissues, including the brain, kidney, muscle, and lungs^14^. This, in turn, can cause toxic side effects such as endocrine dysfunction, growth retardation, and peripheral neuropathies^15,16^. Furthermore, deferiprone and deferasirox have shown significant toxicities (e.g. gastrointestinal bleeding, agranulocytosis, neutropenia, thrombocytopenia, hepatic fibrosis, and kidney failure), which consequently increase drug-induced mortality rate^17–21^. Therefore, there is an unmet need for novel chelation strategies using rationally designed iron chelators that offer improved PK and biodistribution, as well as efficacy and safety.

There have been several attempts to prolong the half-lives to improve iron elimination efficiency and reduce the toxicity by exploiting macromolecules such as dendrimers. However, their biodistribution patterns and elimination pathways are not fully defined (Supplementary Table 2)^16,22–26^. Promoting renal clearance is particularly important in chelation therapy because increased hepatic iron stores in many iron overload patients cause liver dysfunction and limit the capacity of biliary excretion^27^. In contrast, functional impairment in kidney is rare in most iron overload conditions in humans^28^.

Here, we report that renal clearable nanochelators provide favorable biodistribution and PK/PD as well as safety. We design ultrasmall nanochelators that circulate in the blood for a reasonable residence time during which they bind and remove excess iron exclusively via the urinary elimination while bypassing the immune system with negligible nonspecific tissue distribution.

## Results

### Design and synthesis of renal clearable nanochelators (DFO-NPs)

**Fig. 1:**
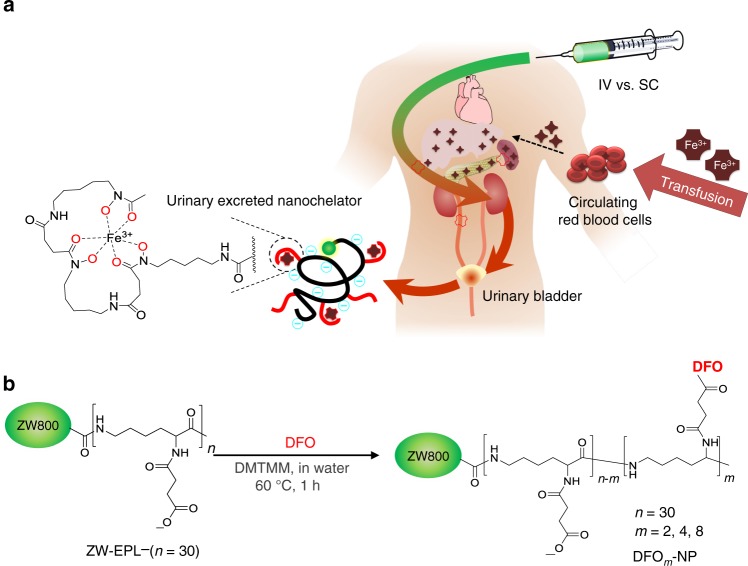
Renal clearable nanochelators to treat secondary hemochromatosis: **a** Nanochelators are composed of multiple DFO moieties conjugated on renal clearable EPL backbone to minimize nonspecific uptake into immune-related organs, while efficiently capturing plasma iron and being cleared exclusively by the kidneys. **b** Synthetic scheme of renal clearable DFO-NPs. *m* represents the number of DFO moieties conjugated on EPL. NIR fluorescent ZW800-1C (green color) was conjugated to track the fate of nanochelators in the body

We have previously demonstrated that physicochemical properties such as size, shape, surface charges, and hydrophilicity/lipophilicity control the fate of inorganic/organic hybrid NPs in the body^29–33^. We proved that zwitterionic NPs with smaller hydrodynamic diameter (HD) than the kidney threshold (<5.5 nm) are preferred for renal clearance^34^. In this study, we designed renal clearable iron nanochelators using ε-poly-l-lysine (EPL) as a biocompatible backbone since EPL is a natural antimicrobial cationic peptide and generally recognized as safe (GRAS) as a food preservative (Fig. 1a)^35^. To track the in vivo behavior of nanochelators, the zwitterionic near-infrared (NIR) fluorophore ZW800-1C^36^ was conjugated to the amine groups of EPL (Supplementary Fig. 1). Then, the rest of primary amines on EPL were converted to carboxylates (Conversion was confirmed by ninhydrin test as shown in Supplementary Fig. 2), followed by conjugation of DFO moieties on the backbone (DFO_*m*_-NPs, where *m* is the number of conjugated DFO per each NP; Fig. 1b). To optimize iron-binding properties of nanochelators, the number of DFO moieties on the backbone (*m*) was controlled to be two to eight. We chose DFO as the iron-binding domain in the nanochelator because it is a conventional iron chelator and a hexadentate siderophore containing three hydroxamate groups that bind iron at 1:1 stoichiometry^37^. Thus, unlike bi- or tri-dentate iron chelators, DFO can completely chelate iron without unoccupied coordination sites, which prevents iron ions from catalyzing the hydroxyl radical formation^11^.

### Physicochemical properties of DFO-NPs

The physicochemical properties of DFO-NPs are summarized in Table 1. The purity of synthesized DFO-NPs was over 94% (Supplementary Fig. 3), confirmed by high-performance liquid chromatography (HPLC) analyses with a size-exclusion column. The number of DFO (*m*) per NP was quantified by two methods: (1) physical: direct absorbance measurement of DFO and proton quantification by nuclear magnetic resonance (NMR) (Supplementary Figs. 4 and 5) and (2) functional: iron-binding assay determined by the absorbance at 430 nm of the DFO–iron complex (Supplementary Fig. 6)^38,39^. The iron-binding stoichiometry values of DFO_2_-NP (2.0), DFO_4_-NP (3.5), and DFO_8_-NP (7.5) were in good agreement with those obtained by the physical stoichiometry method within the acceptable ranges (<10% difference). HDs increased gradually from 5.5 nm (DFO_2_-NP) to 6.5 nm (DFO_8_-NP) measured by gel filtration chromatography (Table 1, Supplementary Fig. 7), which were anticipated due to the increased number of DFO per EPL, but the overall HDs were still within the kidney filtration threshold (6–8 nm)^29,40^, indicating that DFO-NPs can be readily excreted through renal glomerular filtration to urine. To evaluate if the conjugation of DFO to EPL could affect DFO–ironbinding properties, we characterized iron-binding affinities of nanochelators by using the ferrozine competition assay^41^ (Supplementary Fig. 8). The dissociation constant (*K*_D_) of DFO_4_-NP to iron was 3.5-fold (*p* < 0.001, *n* = 3) lower than that of native DFO, suggesting that DFO_4_-NP has a comparable iron-binding affinity to native DFO when normalized to the equimolar concentration of DFO. In contrast, the overall binding affinity of DFO_2_-NP and DFO_8_-NP was decreased by 1000-fold and sevenfold, respectively, compared to DFO_4_-NP, which could be attributed to the intramolecular steric effect of DFOs after conjugation to the EPL polymer. The iron-binding affinity of blank NP was negligible.

**Table 1:** Physicochemical characteristics of DFO-NPs

	MW (Da)	# of DFO/NP^a^	Chelation #^b^	HD (nm)	*K*_D_ (M)
DFO	561	1.0^c^	1.0^c^	–	4.83 × 10^−17^
Blank NP	8000	–	–	5.8	4.79 × 10^−9^
DFO_2_-NP	9100	1.9	2.0	5.5	1.39 × 10^−14^
DFO_4_-NP	10,200	3.6	3.5	5.7	1.37 × 10^−17^
DFO_8_-NP	11,300	8.2	7.5	6.5	9.88 × 10^−17^

*MW* molecular weight, *chelation #* chelation stoichiometry, *HD* hydrodynamic diameter, *K*_*D*_ dissociation constant

^a^Obtained by ^1^H-NMR measurement ^b^Calculated by iron binding assay ^c^Theoretical value of DFO

### Pharmacokinetic study of DFO-NPs

**Fig. 2:**
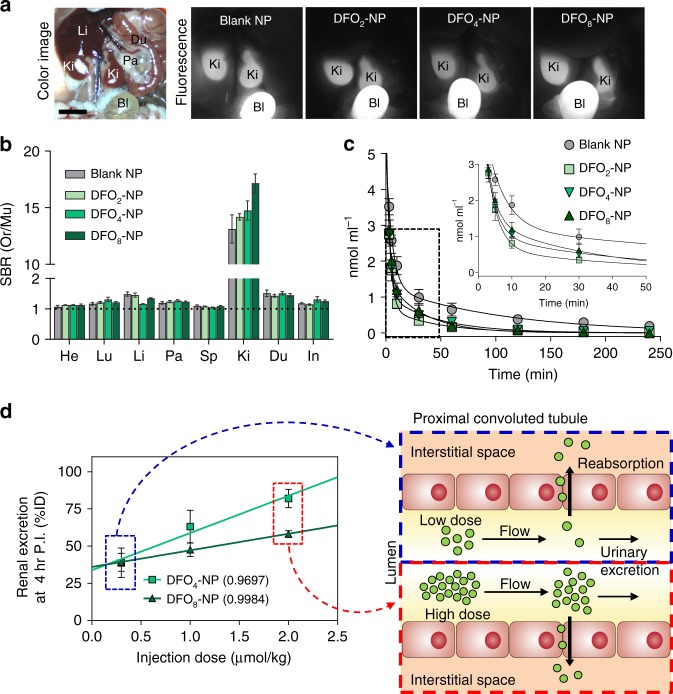
Biodistribution and PK of DFO-NPs. **a** Color and NIR fluorescence images of mouse abdominal cavity. DFO-NPs (0.3 µmol kg^−1^) were IV injected into CD-1 mice 4 h prior to imaging. Bl bladder, Du duodenum, Ki kidney, Li liver, Pa pancreas. Scale bar = 1 cm. **b** Biodistribution of DFO-NPs in major organs resected 4 h post-injection. He heart, In intestine, Lu lung, Mu muscle. The SBR was calculated by the fluorescence intensity of each organ (Or) against Mu (*n* = 5 per group, mean ± SEM). **c** Plasma concentration of DFO-NPs. The concentration (nmol ml^−1^) of DFO-NPs in plasma at each time point was calculated from fluorescence signal intensities. (*n* = 5–8 per group, mean ± SEM). **d** Dose effect on renal clearance efficiency of DFO_4_-NP and DFO_8_-NP ranging from 0.3 to 2 µmol kg^−1^ (*n* = 3 per group, mean ± SEM) (left), and schematic drawing of dose-dependent elimination process at low (right, top) and high doses (right, bottom)

Next, quantitative characterization of the biodistribution and elimination of nanochelators was assessed by performing PK experiments after a single intravenous (IV) injection of DFO-NPs (Fig. 2). For biodistribution, the abdominal cavity was subject to NIR fluorescence imaging at 4 h post-injection (Fig. 2a; NIR optical property is shown in Supplementary Fig. 9). Most DFO-NPs were renally excreted through the kidneys to the urinary bladder, and only negligible amounts of DFO-NPs were found in other organs (Fig. 2b, Supplementary Fig. 10). This result indicates that DFO-NPs can limit the distribution of DFO and likely minimize its side effects in non-target tissues. To quantitatively characterize the PK of nanochelators, plasma concentration–time profiles of DFO-NPs were fitted to the conventional two-compartment PK model. Plasma concentrations of the nanochelators were determined by NIR imaging as shown in Supplementary Fig. 11a. As predicted, all nanochelators disappeared rapidly from blood circulation, as evidenced by the terminal half-lives less than 1.5 h (37–77 min). Interestingly, DFO-NPs were cleared from plasma quicker than blank NPs (Fig. 2c, inset), and the areas under the curves of DFO-NPs were decreased, while the total body clearance values were increased, compared to those of blank NPs. This could be attributed to a decrease in surface charges despite a gradual increase in overall sizes of the DFO-NPs after DFO conjugation.

DFO-NPs also exhibited favorable distribution kinetics (Table 2). First, the volumes of distribution of the central compartment (*V*_1_) for DFO-NPs were 6–9% body weight (i.e. 66-93 ml kg^−1^), consistent with the reported plasma volume for mice^42^. Second, the total volumes of distribution at steady state (*V*_SS_) of DFO-NPs were 19–34% of the body weight, similar to the volume of the extracellular fluids (~20%). These results demonstrate that DFO-NPs immediately circulate in the bloodstream, followed by distribution into the interstitial space, but do not enter the intracellular space, whereas small molecule iron chelators possess large *V*_SS_. For example, evidence indicates that DFO distributes throughout the body; the *V*_SS_ of DFO is total body water in animals^43^ or even greater than total water (1.35 L kg^−1^) in humans^15^. Similarly, the *V*_SS_ of the oral chelator deferiprone is >1 L kg^−1^ (ref. ^44^). These indicate small molecule chelators enter the cells and interact with intracellular components, resulting in the perturbation of cellular function. By contrast, our DFO-NPs allow for the chelation of extracellular iron, which would significantly decrease chelators’ own toxicities. Moreover, since DFO-NP is rapidly cleared from the blood circulation through the kidney, this would eventually create a sink condition such that excess intracellular iron would be mobilized into the plasma without direct chelation inside the cell until there is no chelatable iron in plasma, which increases the safety of iron chelators.

**Table 2:** Pharmacokinetic parameters of DFO-NPs (0.3 µmol kg^−1^)

Pharmacokinetic parameter	Blank NP	DFO_2_-NP	DFO_4_-NP	DFO_8_-NP
*k*_el_ (min^−1^)	0.011 ± 0.003	0.016 ± 0.002	0.019 ± 0.005	0.009 ± 0.001
*t*_1/2α_ (min)	5.72 ± 0.89	2.65 ± 0.66	2.44 ± 0.22	5.66 ± 2.51
*t*_1/2β_ (min)	64.05 ± 24.30	43.00 ± 5.02	36.93 ± 6.89	76.83 ± 11.07
AUC (nmol ml^−1^ min)	189.00 ± 58.52	47.67 ± 7.17	70.71 ± 9.63	59.22 ± 9.13
Clearance (ml min^−1^ kg^−1^)	2.74 ± 1.01	6.99 ± 0.95	4.68 ± 0.67	5.66 ± 1.03
*V*_1_ (ml kg^−1^)	72.86 ± 5.05	68.59 ± 11.85	65.80 ± 9.49	92.65 ± 10.63
*V*_2_ (ml kg^−1^)	115.69 ± 24.37	211.27 ± 39.49	161.65 ± 29.95	249.75 ± 99.32
*V*_ss_ (ml kg^−1^)	188.56 ± 27.60	279.86 ± 45.98	227.45 ± 36.59	342.41 ± 100.51
Urinary excretion (%ID)	54.32 ± 9.50	52.34 ± 13.15	38.73 ± 17.08	39.06 ± 10.51

*k*_el_ elimination rate constant, *t*_1/2α_ distribution half-life, *t*_1/2β_ elimination half-life, AUC area under the curve, *V*_1_ and *V*_2_ volume of distribution in the central compartment and peripheral compartment, respectively, *V*_ss_ volume of distribution at steady state. The time point for urinary excretion is 4 h post-injection. (*n* = 5–8 per group, mean ± SEM)

Based on favorable iron-binding properties, we selected DFO_4_-NP and DFO_8_-NP to carry out dose-dependent biodistribution and PK studies ranging from 0.3 to 2 µmol kg^−1^ (amounts in mg kg^−1^ unit were also provided in Supplementary Table 3). At 4 h post-injection, DFO-NPs did not show any nonspecific biodistribution, even at the highest dose tested (2 µmol kg^−1^), and were exclusively excreted through the urine. No hepatobiliary excretion was detected during the 24 h biodistribution (Supplementary Fig. 12, Movies 1 and 2). The dose–renal excretion (%ID) relationship showed more efficient excretion of DFO_4_-NPs than that of DFO_8_-NPs (Fig. 2d) due to the difference in physicochemical properties including charge-to-mass ratio. Interestingly, after administering high dose of DFO_4_-NPs (2 µmol kg^−1^), renally excreted dose of DFO-NPs proportionally increased in a dose-dependent manner up to 82 %ID. This could be due to the saturation of renal reabsorption of nanochelators at high doses as illustrated in Fig. 2d. In addition, blood clearance results indicate that all nanochelators with different doses cleared rapidly (Supplementary Fig. 13), which could be attributed to high glomerular filtration (200–340 ml per day in mice^45^). Taken together, this indicates that there is a benefit in the kinetics of urinary excretion for DFO-NPs with a higher dose (2 µmol kg^−1^).

### PD and therapeutic efficacy of DFO-NPs

**Fig. 3:**
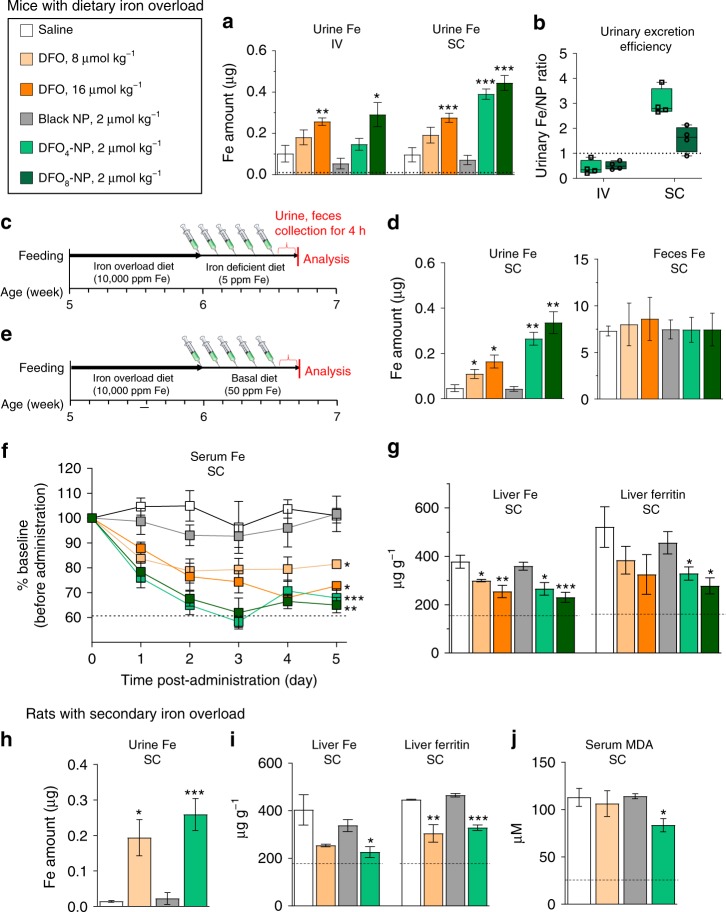
Iron levels in iron overload mice and rats treated daily with DFO-NPs: **a**, **b** Urinary iron excretion and efficiency of DFO-NPs treatment via IV and SC injection compared to saline and DFO treatments at 4 h post-injection. Dotted line in **a** indicates physiological reference levels from CD-1 mice fed basal diet (50 ppm Fe per kg). Dotted line in **b** indicates reference levels for Fe/DFO (centerline, median; whiskers, minimum and maximum). **c** Timeline for an iron-overload mouse model and DFO-NPs treatments for urine and fecal analysis. **d** The amount of iron excreted in urine and feces at 4 h post-injection after chelator treatment shown in **c**. **e** Timeline for an iron overload mouse model and DFO-NPs treatments for serum, liver, and spleen analyses. **f** Serum iron level and **g** iron and ferritin amount in liver of iron-overload mice treated with saline, DFO, and DFO-NPs via SC injection for 5 days. Dotted lines indicate physiological reference levels from CD-1 mice-fed basal diet (50 ppm Fe per kg). **h** Urine iron level, **i** ferritin level in liver, and **j** serum MDA level of iron overload Belgrade rats (*b/b*) treated with saline, DFO, and DFO-NPs via SC injection for 5 days. Dotted lines indicate physiological reference levels from +*/b* rats which display normal iron status. *n* = 3–8 per group, mean ± SEM. Student *t*-test was employed to compare the results between two groups. A one-way ANOVA followed by Tukey’s multiple comparisons test was used to assess the statistical differences among more than two groups. *p* values <0.05 were considered significant: *p < 0.05, **p < 0.01, and ***p < 0.001

We tested the efficacy of DFO-NPs by determining urinary iron excretion in dietary iron overload mice after a single dose of 2 µmol kg^−1^ by IV or SC injection. First, the integrity of the renally excreted DFO-NPs was verified using a molecular weight cutoff (MWCO) filter and HPLC-mass spectroscopy (MS) (Supplementary Fig. 14). No cleaved DFO was found in the urine filtrates, indicating that DFO-NPs are stable after blood circulation and urinary excretion. Next, the iron concentrations were quantified using non-heme iron analysis, and the amounts of the nanochelator were measured by the NIR fluorescence signal intensity using capillary tubes (Supplementary Fig. 11b). After IV injection, iron excretion in the DFO_8_-NP group significantly increased by 2.8-fold (*p* < 0.05) compared to the negative control group injected with saline (Fig. 3a). There was a significant improvement in iron excretion after SC injection in both DFO_4_-NP and DFO_8_-NP groups; the urinary iron excretion was increased by 4.0-fold (DFO_4_-NP) and 4.6-fold (DFO_8_-NP) compared to the saline-injected group. When compared to equimolar administration of native DFO, the nanochelators displayed increased excretion of urinary iron by 2.0-fold (DFO_4_-NP) and 1.6-fold (DFO_8_-NP). These results combined demonstrate that SC injection can be a more favorable injection route for our nanochelators.

Next, we also evaluated the administration route-dependent PK and PD of nanochelators. First, biodistribution results revealed that DFO-NPs after SC injection cleared exclusively into the urinary bladder, very similar to the pattern of IV injection (Supplementary Fig. 15). Second, we compared the relative efficiency of urinary iron excretion between IV and SC injections by dividing the molar amount of urinary iron by that of DFO-NPs (Fig. 3b, Supplementary Table 4). Despite the fact that urinary excretion was 10.7 ± 3.8 %ID (mean ± SEM) after 4 h of SC injection, iron excretion for DFO_4_-NP and DFO_8_-NP after SC injection was more efficient (7.0- and 3.1-fold, respectively) compared with IV injection. This is likely due to the slow release of DFO-NPs after SC injection into the systemic circulation, which increases contact time for DFO-NPs to bind newly released iron from iron-loaded tissues as a result of the redistribution of iron (driven by iron chelation) from intracellular to extracellular spaces; the maximum concentration of DFO-NPs in blood was achieved in 1 h post-injection (Supplementary Fig. 15). In contrast, since IV injection allows for all injected doses immediately available in the bloodstream, some fraction of IV-injected DFO-NPs could be excreted prior to iron binding. Therefore, our results suggest promising applications of nanochelators if coupled with sustainable release systems, such as hydrogels^46^.

The efficacy of DFO-NPs on iron removal was further evaluated after daily SC injections into dietary iron overload mice for 5 days (Fig. 3c). We confirmed that both native DFO and DFO-NPs increased iron excretion in a dose-dependent manner through urine, which is consistent with the result from the single dose injection (Fig. 3d, left). Although it has been reported that native DFO removes excess iron by both biliary and renal pathways^47^, we only found a trend of increased iron excretion via feces, without statistical significance, after DFO treatment (Fig. 3d, right). This is likely due to the substantial amount of iron present in diet. However, compared to the control group, there was no significant change in fecal iron (Fig. 3d) and hepatobiliary clearance of DFO-NPs after DFO-NPs injection (Supplementary Fig. 12). This verifies renal-selective excretion of iron by DFO-NPs. Next, non-heme iron analysis was conducted (Fig. 3e) and the results revealed that DFO_4_-NPs and DFO_8_-NPs decreased serum iron levels to 68% (*p* < 0.005) and 65% (*p* < 0.001), respectively, which were significantly different from 82% (DFO, 8 µmol kg^−1^) and 73% (DFO, 16 µmol kg^−1^) after equimolar DFO administrations (Fig. 3f; serum ferritin level was shown in Supplementary Fig. 16a). Our nanochelators almost completely restored serum iron close to the normal levels as determined in serum from CD-1 mice that were fed normal diets. Furthermore, since the status of ferritin, the major iron-storage protein, is a reliable marker of body iron storage, we quantified ferritin levels by ELISA as well as non-heme iron levels in the liver and spleen of mice after 5-day DFO-NPs exposure. The results showed decreased iron and ferritin levels in the liver (Fig. 3g) and in the spleen (Supplementary Fig. 16b). Additionally, we conducted Prussian Blue staining on organs including liver, spleen, heart, and kidney and estimated iron deposition by image processing in each organ (Supplementary Fig. 17). Values for % area of blue dots significantly decreased in DFO-NPs treated groups, which is consistent with our previous observation of reduced iron and ferritin levels in both liver and spleen of DFO-NPs treated mice. Taken together, our results demonstrate that DFO-NPs efficiently remove excess iron via renal route and decrease body iron to normal levels after repeated administrations.

Due to ineffective erythropoiesis, the homozygous Belgrade rats (*b/b*) display hypochromic, microcytic anemia with high serum iron and hepatic iron loading. The unique feature of iron loading anemia resembles several types of transfusional iron overload, such as thalassemia^48^. We found that SC injections of DFO_4_-NP increased urinary iron contents by 1800% (*p* < 0.05; Fig. 3h), whereas the levels of iron in the liver and spleen were decreased by 44% (*p* < 0.05; Fig. 3i left) and by 32% (*p* < 0.01; Supplementary Fig. 16c, left), respectively, compared to untreated *b/b* rats. Furthermore, DFO_4_-NP treatment decreased the levels of ferritin in the liver and spleen by 26% (*p* < 0.001; Fig. 3i, right) and 12% (*p* < 0.001; Supplementary Fig. 16c, right), respectively, compared to saline-treated controls. Blank NPs did not show any changes. Therefore, our results indicate that DFO-NPs alleviate iron overload conditions resulting from secondary (or non-dietary) iron overload. In addition, since excess iron increases oxidative stress, we further evaluated the levels of malondialdehyde (MDA), a product of lipid peroxidation and a biomarker of oxidative stress, in *b/b* rats. Serum MDA levels were decreased in *b/b* rats by 27% (*p* < 0.05) after DFO_4_-NP treatment compared to saline-treated controls, blank NP, or even equimolar native DFO, suggesting that DFO-NPs would effectively protect the body from iron-induced oxidative damage.

### Toxicity assessment of DFO-NPs

**Fig. 4:**
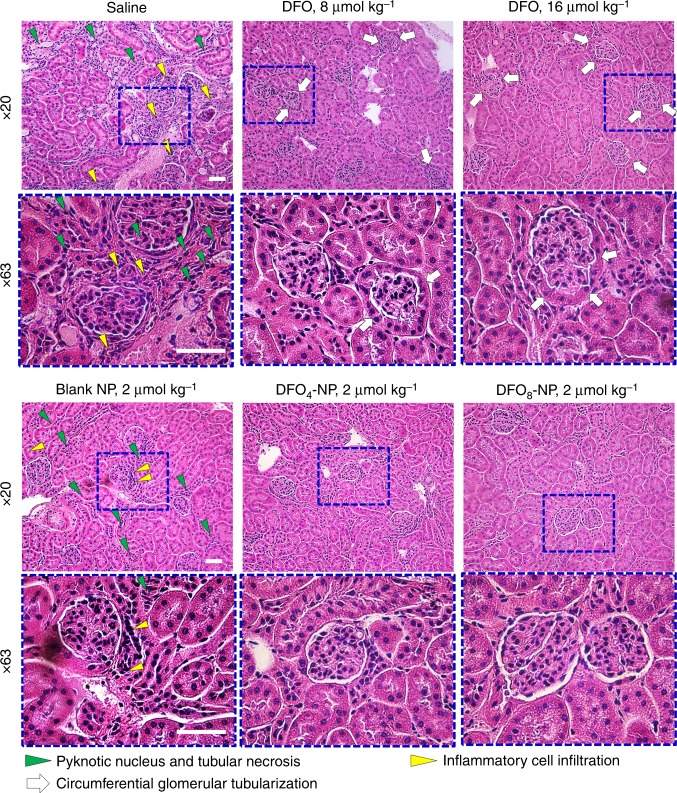
Nephrotoxicity in dietary iron overload mice: H&E-stained sections of renal cortex in mice following 5 days daily treatments of saline, DFO, and DFO-NPs via SC injection. The regions in the blue boxes are magnified at ×63 to show the difference in the preservation of the renal corpuscle architecture. Scale bars: 50 µm

Iron overload is associated with renal injury because iron can undergo the Fenton reaction, accelerating free-radical generation that leads to organelle membrane dysfunction and subsequent cell death^49^. Although chelation therapies with DFO alleviate iron overload-related renal toxicity^50^, long-term and high-dose administration of DFO for chronic iron overload demonstrate nephrotoxicity and renal tubular damage^51,52^. Since DFO-NPs are also cleared by renal filtration, it is important to examine the safety of DFO-NPs on kidney damage after repeated doses. Hence, we evaluated whether repeated administrations of DFO-NPs cause renal dysfunction. We injected native DFO (8 µmol kg^−1^), blank NP (1 µmol kg^−1^), and DFO_8_-NP (1 µmol kg^−1^ as EPL) to CD-1 mice daily by SC injection for 5 days. Another group of CD-1 mice were injected a single dose of DFO-NP (1 µmol kg^−1^) on day 4 for signal comparison (Supplementary Fig. 18). No significant difference in fluorescence intensities was found among those groups, indicating active filtration of DFO-NPs without accumulation or blockage in the kidney. Next, kidney histopathology in dietary iron-overload mice was carried out after daily SC administrations of chelators for 5 days. As shown in Fig. 4, kidney sections stained with hematoxylin and eosin (H&E) of saline-treated iron overload mice demonstrated increased cell swelling, effacement of the brush border, tubular cells with pyknotic nuclei (primarily in the cortex, green arrowheads in Fig. 4), and extensive inflammatory infiltrate (yellow arrowheads), indicative of significant renal injury (H&E staining images for the rest of major organs including liver, spleen, and heart were presented in Supplementary Fig. 19). In contrast, kidney sections of DFOs or DFO-NPs treated mice show reduced infiltrate and tubular necrosis compared to the control group treated with saline. However, a higher dose of native DFO (16 µmol kg^−1^) significantly induced retrograde proliferation of tubular epithelium, leading to the replacement of the parietal glomerular epithelium through a process called glomerular tubularization (white arrows), an indicator of acute kidney injury^53^. In contrast, DFO_8_-NPs with a dose of 2 µmol NP kg^−1^, equivalent to the native DFO dose, notably improved the result by retaining renal corpuscle architecture. This result suggests that DFO-NPs ameliorate iron overload-mediated kidney damage while protecting from kidney injury induced by native DFO. In addition, we determined the levels of serum creatinine and blood urea nitrogen (BUN) because changes in these substances indicate an advancedstage of renal injury^54,55^. However, we did not observe differences in either serum creatinine or BUN among the treatment groups (Supplementary Fig. 20).

Next, we have carried out a single-dose acute toxicity test to determine short-term adverse effects (Supplementary Fig. 21). To minimize the number of animals, we started with LD_50_ value of DFO in a mouse (394 mg kg^−1^, calculated based on the free base form) and subsequently tested with 79 and 16 mg kg^−1^ as medium and low doses, respectively. For DFO_4_-NP administration, we injected DFO-NPs (1500, 300, and 60 mg kg^−1^, respectively) that are equimolar to the DFO alone. All the mice died immediately (*n* = 4) with highest dose of DFO alone (LD_50_), and lower doses were not fatal. In contrast, no abnormal behavior related to toxicity was observed in the DFO-NP injected groups. On the 14th day after the initial injection, blood and major organs (heart, liver, spleen, lung, and kidneys) were collected, and biochemical analyses (lactate dehydrogenase (LDH), aspartate aminotransferase (AST), and aminotransferase (ALT)) and histopathological examination by H&E staining were conducted. The biochemistry tests showed that even lower doses of DFO alone mostly increased the levels of all markers. However, DFO-NPs significantly decreased damages, demonstrating no significant changes in the levels of LDH, AST, and ALT. Histological evaluation after H&E staining showed no evidence of tissue damage, inflammation, or morphological changes in DFO-NP treatment groups (Supplementary Fig. 21e). In contrast, several signs of toxicity, such as perivascular hypercellularity in spleen, inflammatory infiltrate, hyaline casts, and interstitial hemorrhage in kidney; and inflammatory infiltrates, intra-alveolar, and intrabronchial deposits in lung, were observed in the DFO alone group. Taken together, DFO-NP not only improved PK/PD and therapeutic efficacy but also greatly reduced the toxicity of DFO.

## Discussion

Iron chelation therapies with DFO have shown marked decrease in morbidity and mortality of iron overload patients^52^. However, the short half-life of DFO^12,13^ requires repeated injections or continuous infusions, which considerably raises a compliance issue^56^. To overcome these issues, two oral chelators, deferiprone and deferasirox, were developed for clinical use. Although those oral chelators improve systemic circulation time about 6- to 48-fold compared with DFO, they show significant dose-limiting side effects, including gastrointestinal bleeding, agranulocytosis, hepatic fibrosis, and renal failure^18–21^ mainly due to their off-target toxicity^14,15^. In addition, increased iron accumulation in the liver could hamper the hepatobiliary excretion of the deferasirox–iron complex^27^ since the liver is the primary site of iron storage. Therefore, there is an unmet need for a new therapeutic strategy that addresses these PK and efficacy/toxicity concerns.

One strategy is to develop therapeutic NPs, such as dextran-DFO^57^, pentastarch-DFO^58,59^, starch-DFO^60^, hydroxyethyl starch-DFO^61–63^, and dendritic polyglycerol^16,25^ with a focus on increasing circulation of small molecule chelators. Despite such advantages in PK with longer half-lives^11^, these formulations have several drawbacks, including potential immune response and safety^62^. In contrast, we aimed to develop nanochelators with reasonable blood circulation and rapid renal excretion. This idea is based on our previous work on renal clearable NPs, where the physicochemical properties of inorganic/organic hybrid NPs govern their fate in the body^29–33^. We also confirmed that polymeric NPs follow this rule by distributing into the extracellular fluid, followed by preferential excretion through the kidneys owing to their small size (<5.5 nm) and balanced charges^34^. These characteristics significantly reduce nonspecific uptake into major organs and decrease the potential toxicity of NP-based pharmaceuticals.

In this view, our nanochelators have three key features. First, while increased hepatic iron stores in many iron overload patients deteriorate liver function and limit the capacity of biliary excretion^27^, which subsequently restricts the clearance of chelators, our nanochelators circumvent these drawbacks. To the best of our knowledge, there has not been a single peer-reviewed paper that describes renal-selective polymeric iron chelators. Thus, our renal clearable nanochelation therapy could avoid iron overload-related pathophysiological complications in the liver and improve abnormal body iron status.

Second, our nanochelators provide favorable PK/PD, which allows chelation of extracellular iron and decreases toxicities significantly. Ideal chelators should be cleared as soon as they bind to iron due to their potential systemic accumulation and tissue toxicity, while maintaining reasonable systemic circulation. Thus, clinical efficacy would be greatly improved if our renal-selective nanochelators are coupled with controlled release formulations, which would result in long-acting nanochelators (as opposed to long-circulating chelators). As such, we are currently evaluating hydrogel-based sustained release systems to improve the chelation efficacy of nanochelators after SC administration. Considering the need for lifelong administrations of the chelators in patients with iron overload, hydrogel-based nanochelators offer significant advantages over the current chelation therapies.

Third, our nanochelators significantly improve safety. In the single-dose acute toxicity results, even the highest dose (1500 mg kg^−1^) in nanochelators treatment group yielded no evidence of tissue damage, inflammation, or morphological change in both of histological and biochemical analyses. On the contrary, DFO alone (even lower doses) showed several signs of toxicity, such as perivascular hypercellularity in the spleen, inflammatory infiltrate, hyaline casts, and renal interstitial hemorrhage in the kidney, and intra-alveolar and intrabronchial deposits in the lung.

In summary, we have designed a new type of iron nanochelator comprising the FDA-approved DFO and renal clearable polymeric NPs. The nanochelator has improved PK/PD with reasonable half-life and renal-selective elimination of excessive iron. In addition, repeated SC injections of nanochelators improved urinary iron excretion, and the body iron status was restored to normal levels in both dietary iron overload mice and secondary iron overload rats. More importantly, systemically circulating nanochelators reduced iron toxicity dramatically without showing any signs of DFO-induced nephrotoxicity, even at high doses. Together, our renal clearable nanochelators greatly improve therapeutic efficacy and mitigate chelator-associated adverse effects, which provides a promising clinical advancement for safer iron chelation therapy in patients who suffer from iron overload disorders.

## Methods

### Reagents and materials

Epsilon-poly-l-lysine (EPL; MW ~4000) was supplied by Wilshire Tech Inc. (Princeton, NJ). Succinic anhydride (SA), dipyrrolidino(*N*-succinimidyloxy)carbenium hexafluorophosphate (HSPyU), 4-(4,6-dimethoxy-1,3,5-triazin-2-yl)-4-methyl-morpholinium chloride (DMTMM), deferoxamine mesylate (DFO), bovine serum albumin (BSA), diisopropylethylamine (DIEA), ninhydrin, sodium acetate, sodium hydroxide (NaOH), mercaptoacetic acid, bathophenanthrolinedisulfonic acid (BPS), ferric chloride, trichloroacetic acid (TCA), conc-HCl (12.1 M), acetone, ethyl acetate (EA), and ethanol (EtOH) were purchased from Fisher Scientific (Pittsburgh, PA), Sigma-Aldrich (Saint Louis, MO), or Acros Organics (Morris Plains, NJ), and used without further purification.

### Synthesis of ZW800-1C-conjugated EPL

ZW800-1C, a zwitterionic NIR fluorophore emitting 800 nm fluorescence, was used in this study as an imaging modality^64^. ZW800-1C NHS active ester was prepared by using HSPyU and the reactant was dissolved in DMSO (25.5 mg ml^−1^). Fifty micromoles of ZW800-1C NHS ester stock solution (2 ml) was added dropwise to the EPL (200 mg; 50 µmol) solution in PBS (pH 8.0, 20 ml) with vigorous stirring. After 3 h, the reaction mixture was precipitated by adding excess acetone/EA (4/1, v/v; >200 ml) for purification. The reactant was centrifuged at 4000 × *g* for 10 min, and the supernatant was discarded. The resulting pellet was resuspended in water (<2.0 ml) and reprecipitated with acetone/EA (4/1, v/v; 50 ml). The precipitated pellet was washed with EtOH and acetone several times. After the final washing with acetone, the reactant was dried under vacuum. To convert amines to carboxylates, ZW800-1C-conjugated EPL (ZW-EPL) was dissolved in 25 ml of PBS (pH 8.0) and 1.8 ml of SA stock solution (250 mg ml^−1^ in DMSO) was added with stirring at room temperature. The pH of reaction mixture was maintained to be 7.0 by adding 6 N NaOH solution. After 30 min reaction, the conversion of amines to carboxylates was 98.3%, confirmed by the ninhydrin test (Supplementary Fig. 2)^34^.

### Synthesis of renal clearable nanochelator

DFO solution (55 µmol; 36 mg in 360 µl of water, pH neutralized by adding of NaOH solution) were added to ZW-EPL^−^ solution (2 µmol; 16 mg in 1 ml of water) in the presence of DMTMM (15 mg, 150 µmol; 30 mg, 300 µmol; 45 mg, 450 µmol) to produce DFO_2_-NP, DFO_4_-NP, and DFO_8_-NP, respectively. The reactant was stirred vigorously at 60 °C for 1 h, followed by adding excess acetone/EA (4/1, v/v; >200 ml) for purification. The reaction mixture was centrifuged, resuspended, and washed with acetone/EA (4/1, v/v; 50 ml) several times. After the sample was completely dried under vacuum, it was redissolved in water (0.5 ml) for further purification using gel fraction chromatography (GFC; AKTA Purifier; GE Healthcare) equipped with a polyacrylamide P-6 column (Bio-Rad). Sample fractions were collected with monitoring absorbance and conductivity. The final product was lyophilized and stored at −20 °C until use.

### Size-exclusion chromatography analysis

The purity of nanochelater was measured using size-exclusion chromatography (SEC) on the Waters HPLC system consisting of a 1260 binary pump with a 1260 ALS injector, a 35900E photodiode array (PDA) detector 210–800 nm, a 2475 multi-wavelength fluorescence detector (Waters, Ex 770 nm and Em 790 nm), and an ACQUITY QDa MS detector (Waters). A portion of the eluent flowed into the PDA equipped with an Ultrahydrogel2000 (7.8 × 300 mm; Waters) SEC column. Mobile phase was isocratic with 0.1% formic acid in water for 30 min at a flow rate of 0.75 ml min^−1^. To measure hydrodynamic diameter of DFO-EPLs, GFC was performed using the AKTA Purifier (GE healthcare) with a Superose6 10/300 GL column (GE Healthcare). Calibration standard of hydrodynamic diameter was obtained by injecting 100 µl of protein standards containing ribonuclease (12.7 kDa, 3.2 nm), ovalbumin (44 kDa, 6.1 nm), aldolase (158 kDa, 9.6 nm), and ferritin (440 kDa, 12.2 nm) to GFC with the same mobile phase and flow rate. Partition coefficient, *K*_av_ was obtained by the following equation: *K*_av_ (*V*_e_−*V*_0_)/(*V*_c_−*V*_0_), where *V*_0_, *V*_c_, and *V*_e_ are column void volume, geometric column volume, and eluent volume, respectively.

### Physical and functional stoichiometry of nanochelators

To calculate the physical stoichiometry of DFO moieties on EPL, a series of standard DFO solutions was prepared and their absorbance was measured at 200 nm. The changes in absorption spectra of ZW-EPL^−^ before and after DFO conjugation were measured, and the number of DFO moieties on the EPL backbone was calculated based on the Beer–Lambert law, where the extinction coefficient of DFO at 200 nm is 0.025 µM^−1^ cm^−1^. In addition to the physical stoichiometry, the number of DFOs on succinylated EPL was confirmed by ^1^H-NMR measurement using peak integration values of succinic acid’s protons at 2.4 ppm and DFO’s protons at positions 6 and 13 at 2.7 ppm (Supplementary Fig. 5). On the other hand, the functional stoichiometry was calculated based on the absorption value of Fe(III) pre DFO complex at 430 nm, which increased with the degree of Fe(III) pre DFO complex formation. The titration curve was prepared by adding Fe(III) chloride solution (5 µM) in the solution of DFO-NPs (20 µM) with continuous measurement of the absorbance change at 430 nm (Supplementary Fig. 6)^38,39^.

### Estimation of dissociation constant (*K*_D_) of nanochelators

FeCl_3_ (25 µM) was pre-mixed with ferrozine (75 µM; Sigma-Aldrich) in a 25 mM MES/Tris buffer at pH 7.0 containing 62.5 mM sodium ascorbate (Sigma-Aldrich). Then, pre-calculated amounts of blank NP, DFO_2_-NP, DFO_4_-NP, and DFO_8_-NP were mixed with Fe(III). The solutions were allowed to equilibrate for 24 h before measuring the absorbance at 562 nm (Supplementary Fig. 7). The EC_50_ values for each chelator were calculated by four-parameter logistic regression (dotted curves) using SigmaPlot (ver. 12.3; Systat Software INC., Sar Jose, CA) after plotting the absorbance vs. chelator concentrations, and the dissociation constant *K*_D_ for each complex was determined using the equation:

$$K_{D,\, ligand} = \frac{[{\mathrm{ligand}}]} {{K_{A},\, {competitor}} \times [{\mathrm{EC}}_{50}]},$$

where *K*_A_ is an association constant, the ligand is the molecule originally bound to iron (i.e. ferrozine), and the competitor is the competing chelator (DFO alone, blank NP, or DFO-NP)^41,65,66^.

### Biodistribution and PK of nanochelators

Animals were housed in an AAALAC-certified facility and studied under the supervision of MGH IACUC in accordance with the approved institutional protocol (#2016N000136). Six-week-old CD-1 mice (male; 25–30 g; Charles River Laboratories, Wilmington, MA) were maintained under general anesthesia by inhalation of isoflurane or intraperitoneal injection with 100 mg kg^−1^ ketamine and 10 mg kg^−1^ xylazine (Webster Veterinary, Fort Devens, MA). The penis was ligated to collect the full amount of urine. Mice were injected with of blank NP or each DFO-NP (0.3, 1, and 2 µmol as NP kg^−1^) in 5 wt/v% BSA containing saline, and blood was sampled by cutting the end of the tail at the following time points: 0 (before DFO-NP administration to serve as reference), 1, 3, 5, 10, 30, 60, 120, 180, and 240 min. Blood was stored in an ice box to prevent clotting. Pharmacokinetic analysis was performed using the two-compartment model (or bi-exponential decay) to estimate pharmacokinetic parameters, including clearance, volume of distribution and blood half-life. After 4 h post-injection, mice were sacrificed to image organs and urine samples collected from the urinary bladder. At least three mice were analyzed for each group. Mice were imaged using the in-house built real-time intraoperative NIR imaging system (K-FLARE). A 760 nm excitation laser source (4 mW cm^−2^) was used with white light (400–650 nm; 40,000 lux), and color and NIR fluorescence images were acquired simultaneously with customized software. The collected blood samples were centrifuged at 2000 × *g* for 20 min to separate plasma and blood cells, and the supernatants were filled into capillary microtubes (Fisher Scientific, Pittsburgh, PA). Collected urine samples were also filled into capillary microtubes. Fluorescence intensities of the microtubes were measured by the NIR imaging system along with a set of standard DFO-NP samples of known concentrations (Supplementary Fig. 11).

### Urinary excretion of iron after a single dose injection

Animals were housed in an AAALAC-certified facility and were studied under the supervision of Northeastern University IACUC in accordance with the approved institutional protocols (16-0305R/18-0310R). CD-1 mice (male; 30-35 g; Charles River Laboratories) were fed high-iron diet (10,000 ppm Fe per kg) for one week and administered with saline, blank NP (2 µmol kg^−1^), DFO alone (8 or 16 µmol kg^−1^), DFO_4_-NP (2 µmol as NP kg^−1^) or DFO_8_-NP (2 µmol as NP kg^−1^) by IV or SC injection. The penis was ligated to collect the full amount of urine, which was collected 4 h post-injection after euthanasia. Non-heme iron levels in urine were quantified by colorimetric analysis using BPS.

### Excretions of iron after repeated doses

CD-1 mice (male; 30-35 g; Charles River Laboratories) were fed high-iron diet (10,000 ppm Fe per kg) for 1 week and then fed iron-deficient diet (5 ppm Fe per kg) for a week, during which the mice were administered with saline, blank NP (2 µmol kg^−1^), DFO (8 or 16 µmol kg^−1^), DFO_4_-NP, or DFO_8_-NP (2 µmol as NP kg^−1^) by SC injection twice a day for 5 days. The penis was ligated to collect the full amount of urine at latest injection. At 4 h post-injection from the latest treatments, the mice were sacrificed and urine and feces were collected for iron concentration analysis.

### Iron chelation efficacy of DFO-NP after repeated doses

CD-1 mice (male; 30–35 g; Charles River Laboratories) were fed high-iron diet (10,000 ppm Fe per kg) for 1 week. Thereafter, all mice were treated with basal diet and administered with saline, blank NP (2 µmol kg^−1^), DFO (8 or 16 µmol kg^−1^), DFO_4_-NP, or DFO_8_-NP (2 µmol as NP kg^−1^) by SC injection twice a day for 5 days. A separate cohort of CD-1 mice fed basal diet (50 ppm Fe per kg) was included to provide physiological reference levels of iron. At 24 h after the last dose, mice were sacrificed, followed by a collection of blood, heart, liver, spleen, and kidneys for the analyses of iron, ferritin, and histology.

### Iron chelation efficacy in a rat model after repeated doses

Heterozygous (*+/b*) and homozygous (*b/b*) Belgrade rats (Fischer F344 background) were provided from in-house breeding colonies^67^. Female rats (9–11 weeks old) were given an iron-supplemented diet containing 500 mg of iron per kg (TD.02385; Harlan Teklad, Madison, WI) to support anemic condition of *b/b* rats^68^. Rats were administered with blank NP (2 µmol kg^−1^), DFO (8 µmol kg^−1^), or DFO_4_-NP (2 µmol as NP kg^−1^) by SC injection twice a day for 5 days. The +*/b* rats which display normal iron status were included to provide physiological reference levels of iron. On day 6, rats were administered with one more dose and urethral closure was performed to collect the full amount of urine. Four hours after the last dose, rats were sacrificed, followed by a collection of urine, blood, liver, and spleen. The levels of MDA were determined in serum samples using assay kit (Cayman Chemical).

### Iron analysis

Tissues (liver and spleen) were digested in an acid solution (10% trichloroacetic acid and 3 M HCl) at 65 °C for 20 h. Non-heme iron concentrations in tissues were quantified by a colorimetric assay with BPS measuring the absorbance at 535 nm. Urine was digested in the acid solution at 65 °C for 1 h, followed by centrifugation at 5000 × *g* for 5 min. Urine and serum iron was determined by BPS-based colorimetry with background correction.

### Ferritin analysis

Serum was harvested from blood. Tissues (liver and spleen) were homogenized in RIPA buffer (1:10 time dilution; w/v). The lysate was centrifuged at 16,000 × *g* for 6 min at 4 °C and the supernatant was collected. Serum and tissue supernatant were diluted by diluent buffer and subjected to mouse ferritin ELISA (Abcam ab157713) or rat ferritin ELISA (Abcam ab157732) according to the manufacturer’s instructions.

### Single dose acute toxicity test of DFO-NP

Six-week-old CD-1 mice (male; 25-30 g) were intravenously injected with 394 (LD_50_ of DFO), 79, and 16 mg kg^−1^ of DFO alone. 1500, 300, and 60 mg kg^−1^ of DFO-NP_4_ that are equimolar to the DFO alone were injected. A body weight, clinical signs (e.g. abnormal gait and posture or muscle tone), and mortality were monitored for 2 weeks. After that, all mice were sacrificed, followed by a collection of blood, heart, liver, spleen, and kidneys for the analyses of biochemical markers and histology.

### Quantitative and statistical analyses

The fluorescence and background intensities of a region of interest over each tissue were quantified using customized imaging software and ImageJ v1.51j8 (National Institutes of Health, Bethesda, MD). The signal-to-background ratio (SBR) was calculated as SBR = fluorescence/background, where the background is the fluorescence intensity of the muscle. All data depict the mean ± SEM or mean ± standard deviation (s.d.) with a minimum of three biological replicates. Student *t*-test was employed to compare the results between two groups. A one-way *ANOVA* followed by Tukey’s multiple comparisons test was used to assess the statistical differences among more than two groups. *p* values <0.05 were considered significant: *p < 0.05, **p < 0.01, ***p < 0.001, and ****p < 0.0001.

### Reporting summary

Further information on research design is available in the Nature Research Reporting Summary linked to this article.

## Supplementary information

Supplementary Information

Description of additional Supplementary Files

Supplementary Movie 1

Supplementary Movie 2

Reporting Summary

## Data Availability

The data that support the findings of this study are available from the authors on reasonable request, see author contributions for specific data sets.
