# Supplementary material for: Renal clearable nanochelators for iron overload therapy
Source: Nat Commun. 2019 Nov 13;10:5134. doi: 10.1038/s41467-019-13143-z (PMC6853917; doi:10.1038/s41467-019-13143-z)
Supplement: Supplementary file 1 — Supplementary Information [file 41467_2019_13143_MOESM1_ESM.pdf]

Supplementary Information

Renal Clearable Nanochelator for Iron Overload Therapy

Homan Kang,[1#] Murui Han,[2#] Jie Xue,[2] Yoonji Baek,[1] JuOae Chang,[2] Shuang Hu,[1]
HaYoung Nam,[2] Min Joo Jo,[1] Georges El Fakhri,[1] Michael P. Hutchens,[3,4]
Hak Soo Choi,[1,]* and Jonghan Kim[2,]*

[1]Gordon Center for Medical Imaging, Department of Radiology, Massachusetts General Hospital and Harvard Medical School, Boston, MA 02114, USA
[2]Department of Pharmaceutical Sciences, Northeastern University, Boston, MA 02115, USA
[3]Anesthesiology and Perioperative Medicine, Oregon Health & Science University, Portland, OR 97239, USA
[4]Portland Veterans Affairs Medical Center, Portland Oregon, 97239, USA

# These authors contributed equally to this work

*Correspondence to: HSC at hchoi12@mgh.harvard.edu or JK at j.kim@neu.edu

This file includes:

Supplementary Table 1. Properties of FDA-approved iron chelators.
Supplementary Table 2. Summary of macromolecule-based iron chelators.
Supplementary Table 3. Conversion of DFO-NP concentration to mg/kg.
Supplementary Table 4. The amount of iron and DFO-NP excretion and molar ratio of iron/DFO-NP.
Supplementary Figure 1. Synthetic routes of ZW800-1 conjugation on EPL, followed by succinylation.
Supplementary Figure 2. Ninhydrin test of ZW-EPL samples.
Supplementary Figure 3. Retention time profiles of DFO-NPs by HPLC analyses.
Supplementary Figure 4. Physical stoichiometry between DFO and EPL.
Supplementary Figure 5. ^{1}H NMR spectra of DFO, Blank NP, and DFO-NPs in D_2O.
Supplementary Figure 6. UV-vis absorption spectra of Fe(III)-titrated DFO-NPs.
Supplementary Figure 7. GFC profiles of DFO-NPs to estimate hydrodynamic diameter (HD).
Supplementary Figure 8. Competitive iron binding assay to estimate iron binding affinity of DFO-NPs.
Supplementary Figure 9. Representative absorbance and fluorescence spectra of DFO-NPs in water.
Supplementary Figure 10. Color and NIR fluorescence images of dissected organs.
Supplementary Figure 11. Representative NIR images of serum and urine samples.
Supplementary Figure 12. Evaluation of excretion route of DFO-NPs.
Supplementary Figure 13. Dose dependency on biodistribution PK of DFO-NPs.
Supplementary Figure 14. Purification and analyses of collected urine samples.
Supplementary Figure 15. PK and biodistribution of DFO-NPs post-SC injection.

Supplementary Figure 16. Serum ferritin, spleen iron, and ferritin levels of iron overload mice and rats.

Supplementary Figure 17. Prussian blue-stained sections of organs.

Supplementary Figure 18. Color and NIR images of kidney sections from CD-1 mice.

Supplementary Figure 19. H&E-stained sections of liver, spleen, and heart.

Supplementary Figure 20. Biochemical analysis of DFO-NPs in iron-overload animals.

Supplementary Figure 21. Single dose acute toxicity test; biochemical analysis and tissue histology.

Supplementary Movie 1. Real time movie of mouse abdominal injected with DFO_4-NP corresponding to Supplementary Figure 12a.

Supplementary Movie 2. Real time movie of mouse abdominal injected with DFO_8-NP corresponding to Supplementary Figure 12b.

Supplementary Tables

Supplementary Table 1 General properties of FDA-approved iron chelators

	Deferoxamine (Desferal)	Deferasirox (Exjade)	Deferiprone (Ferriprox, L1)
Dose (mg kg^{-1} day^{-1})	30-60	20-30	75-100
Stoichiometry (chelator:iron)	Hexadentate (1:1)	Tridentate (2:1)	Bidentate (3:1)
Dose route	IV, SC	Oral	Oral
Bioavailability	Negligible	70%	40%
Half-life in humans	20 min	8-16 h	2-4 h
Elimination route	Urine, Fecal	Fecal (Urine <8%)	Urine
Adverse effects	Sensorineural hearing loss, visual electroretinographic disturbances, impaired growth, bone development	Gastrointestinal bleeding, renal toxicity, hepatic dysfunction, thrombocytopenia	Agranulocytosis, neutropenia, infection, arthralgia, elevated liver enzymes

Supplementary Table 2 Summary of polymeric nanoparticle-based iron chelators and their excretion routes

Type	HD (nm)	Urinary excretion (%ID)	Hepatobiliary excretion (%ID)	Iron excretion in urine[a]	Iron excretion in feces[a]	Ref.
Small size EPL	5.7	82.04 at 4h	<0.2 at 24h	Y	N	This study
Small size EPL	6.5	58.10 at 4h	<0.2 at 24h	Y	N	
Degradable nanogel	240	N/P	N/P	Y	Y	1
Nanogel	36	N/P	N/P	Y	Y	2
	101	N/P	N/P	Y	Y	
Small size melanin	7.0	N/P	N/P	Y	Y	3
Dendrimer	5.4	8.92 at 144 h	43.92 at 144 h	N/P	N/P	4
	9.8	4.76 at 144 h	6.45 at 144 h	N/P	N/P	
	15.4	8.77 at 144 h	8.18 at 144 h	Y	N/P	
Degradable macromolecule	3.5	N/P	N/P	Y	Y	5
Degradable nanogel	136	N/P	N/P	N/P	N/P	6
Dendrimer	9.8	8.77 at 144 h	8.18 at 144 h	N/P	N/P	7
	11.2	N/P	N/P	Y	Y	
	15.4	4.76 at 144 h	6.45 at 144 h	N/P	N/P	
Degradable macromolecule	N/P	N/P	N/P	N/P	N/P	8

[a]N/P: not provided; Y: significance; N: no significance

Supplementary Table 3 Conversion of DFO-NP concentration to mg kg^{-1}

	Convert to mg kg^{-1}			Conversion factor
	0.3 μmol kg^{-1}	1 μmol kg^{-1}	2 μmol kg^{-1}	
Blank NP	2.4 mg kg^{-1}	8 mg kg^{-1}	16 mg kg^{-1}	8
DFO_2-NP	2.73 mg kg^{-1}	9.1 mg kg^{-1}	18.2 mg kg^{-1}	9.1
DFO_4-NP	3.06 mg kg^{-1}	10.2 mg kg^{-1}	20.4 mg kg^{-1}	10.2
DFO_8-NP	3.39 mg kg^{-1}	11.3 mg kg^{-1}	22.6 mg kg^{-1}	11.3

Supplementary Table 4 The amount of iron and DFO-NP excretion and molar ratio of iron/DFO-NP at 4 h post-injection (SC and IV) (n=5, mean ± SEM)

Injection route	Excretion	DFO_4-NP	DFO_8-NP
IV injection	Urinary excreted Fe (nmol)	4.92±0.42	6.07±1.27
	Urinary excreted DFO-NP (nmol)	14.66±3.72	12.18±2.86
	Fe/NP ratio	0.43±0.14	0.51±0.08
SC injection	Urinary excreted Fe (nmol)	8.98±0.2.44	8.786±1.108
	Urinary excreted DFO-NP (nmol)	3.24±0.843	4.87±0.42
	Fe/NP ratio	2.75±0.06	1.81±0.18

Supplementary Figures

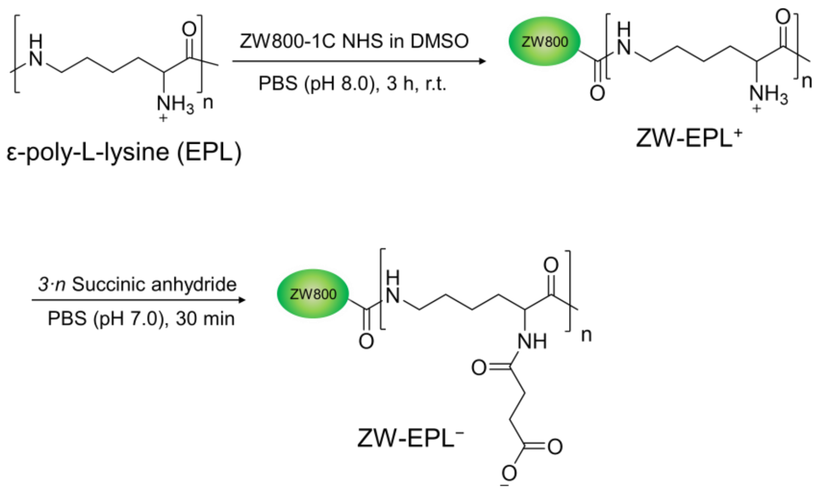

Supplementary Figure 1. Synthetic routes of ZW800-1 conjugation on EPL, followed by succinylation.

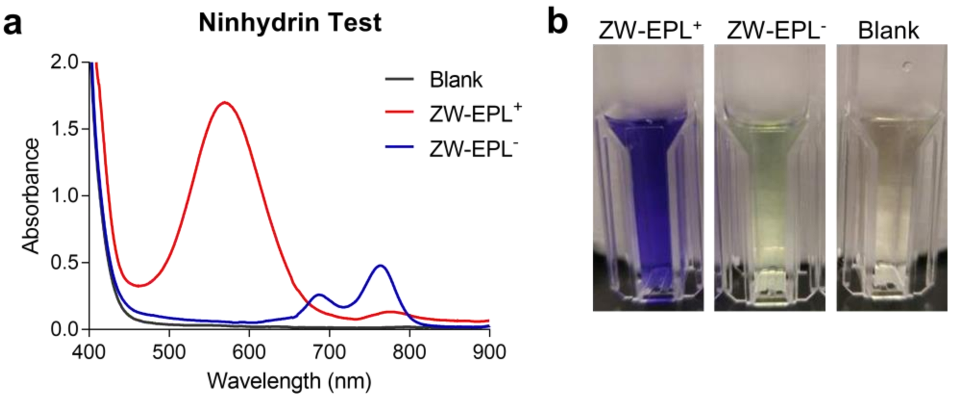

Supplementary Figure 2. Ninhydrin test of ZW-EPL samples: (a) absorbance and fluorescence spectra of DFO-NPs in water (20 μM). (b) Photos of each sample. Green color (ZW-EPL^{-}) represents the existence of ZW800-1, while purple color (ZW-EPL^{+}) indicates the formation of Ruhemann's purple produced by free amines reaction with ninhydrin.

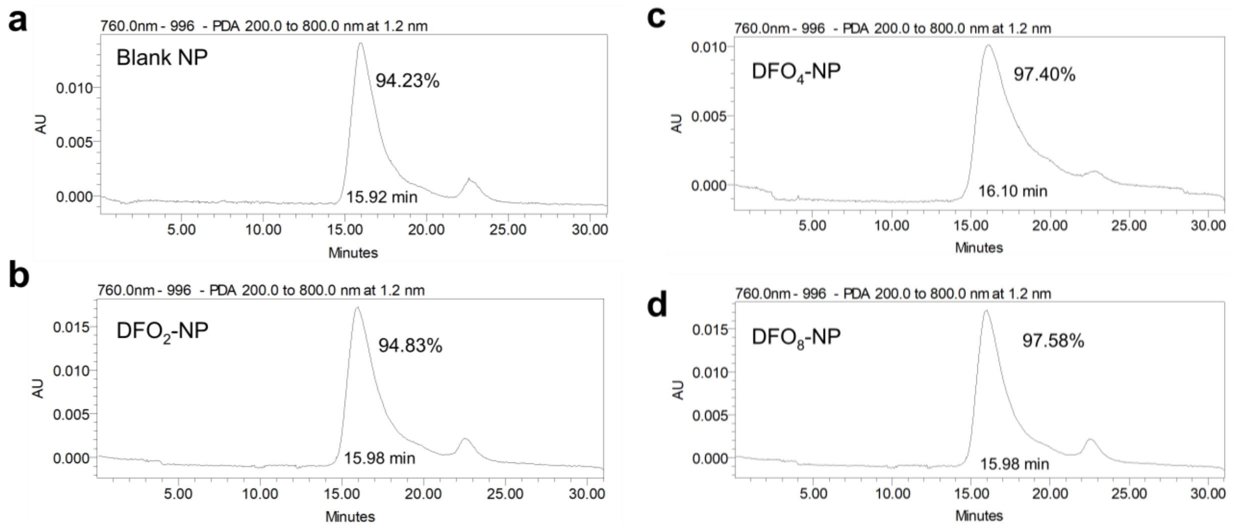

Supplementary Figure 3. Retention time profiles by HPLC analyses: (a) Blank NP, (b) DFO_2-NP, (c) DFO_4-NP, and (d) DFO_8-NP.

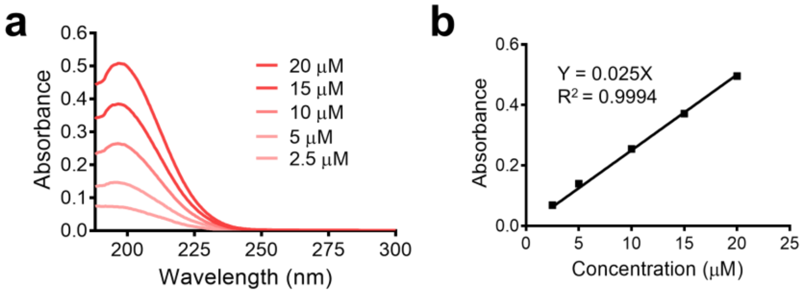

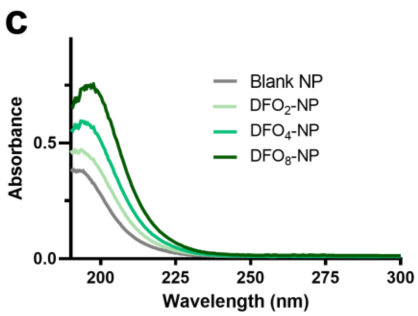

d

Sample (2 μM)	Absolute Abs @ 200 nm	Calculated DFO Conc. (μM)	Stoichiometry (DFO:NP)
Blank NP			
DFO_2-NP	0.101	4.04	**2.0**
DFO_4-NP	0.231	9.24	**4.6**
DFO_8-NP	0.424	16.96	**8.5**

Supplementary Figure 4. **Physical stoichiometry between DFO and EPL:** (a) UV absorption spectra of standard DFO solutions with concentrations ranged from 2.5 to 20 μM. (b) Standard curve fitting with the linear regression of concentration vs. absorbance of DFO standards. (c) UV absorption spectra of DFO-NPs. (d) Physical stoichiometry calculated by using the linear regression plot shown in **b**.

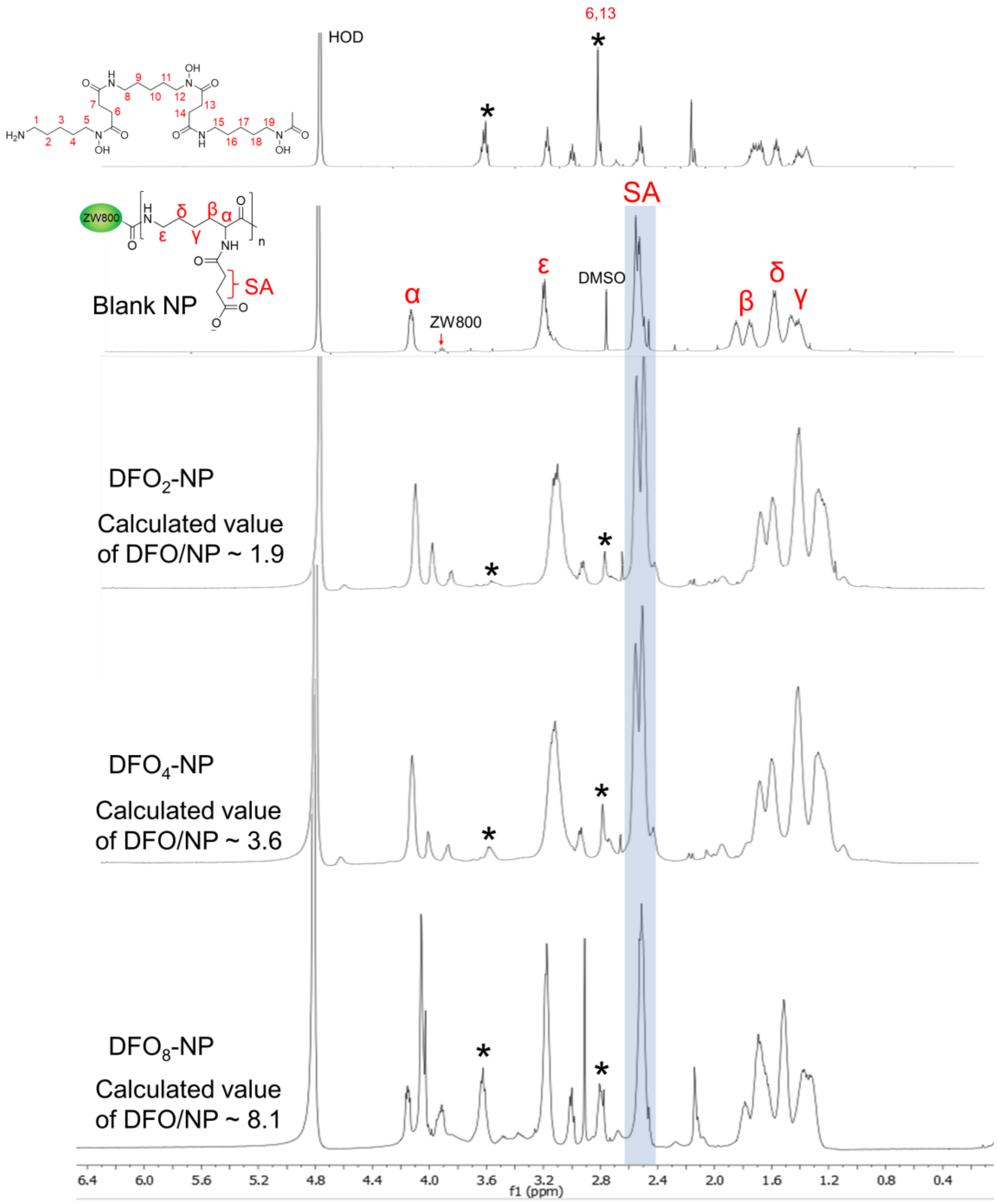

Supplementary Figure 5. ^{1}H-NMR spectra of DFO, blank NP, and DFO-NPs in D_2O. DFO to NP ratio was calculated by integrating of hydrogen atoms of DFO (positions 6,13 at 2.7 ppm, asterisk) and succinic acid (2.4 ppm, sky blue bar). The calculated values of DFO per chain were based on the assumption of 30 lysine units per entire blank NP.

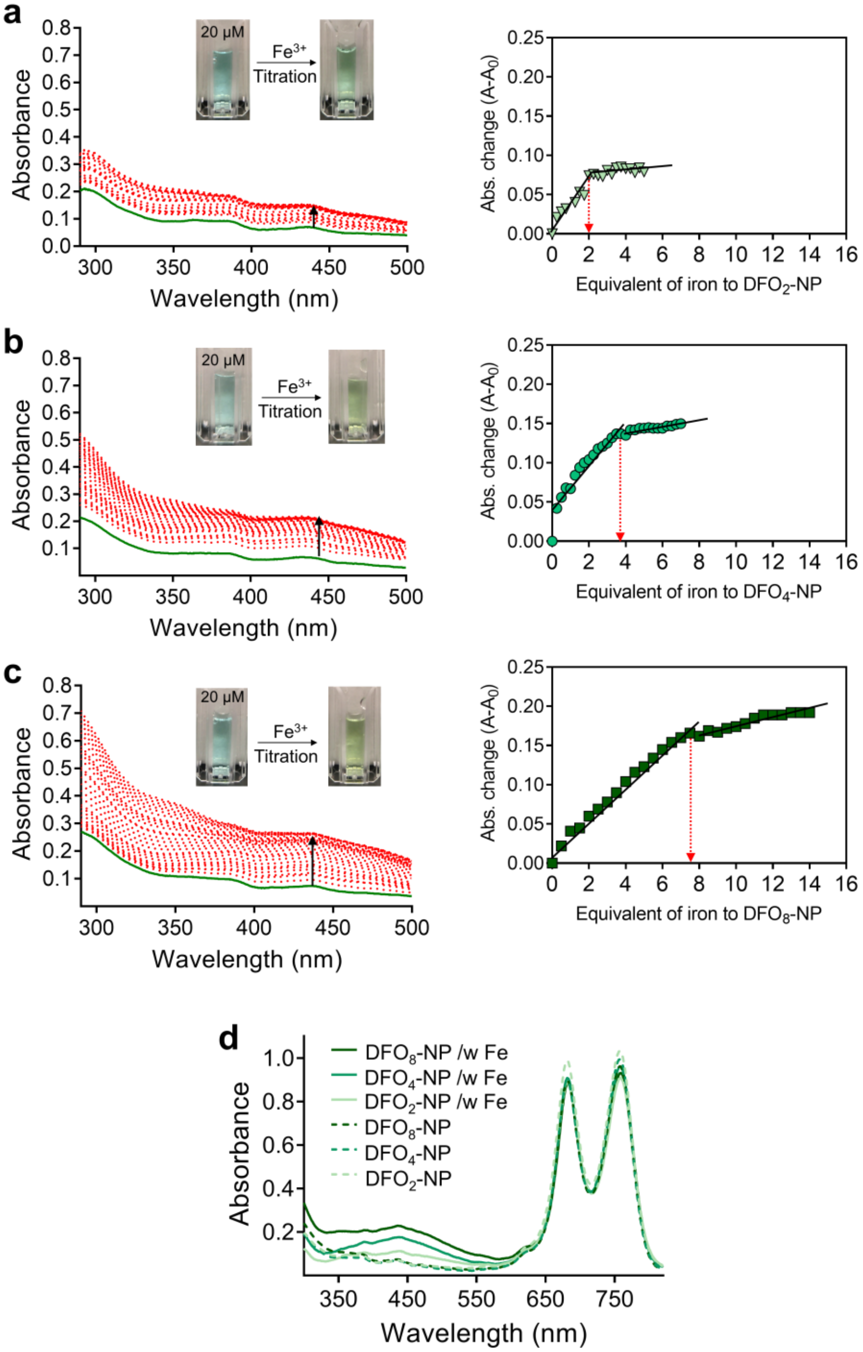

Supplementary Figure 6. UV-vis absorption spectra of Fe(III)-titrated DFO-NPs (red lines in left panel) and titration curves of absorbance changes ($A-A_0$) versus equivalent of iron to each DFO-NP (right panel): (a) DFO$_2$-NPs, (b) DFO$_4$-NPs, and (c) DFO$_8$-NPs. Green lines indicate blank DFO-NP solutions before adding iron solution. Insets: Photos of DFO-NP solutions before and after titration with iron solution. Dotted arrows indicate inflection points where nearly saturated concentrations of iron binding each DFO-NP (20 μM). (d) Absorbance spectra of iron in DFO-NP after removing background iron absorptions.

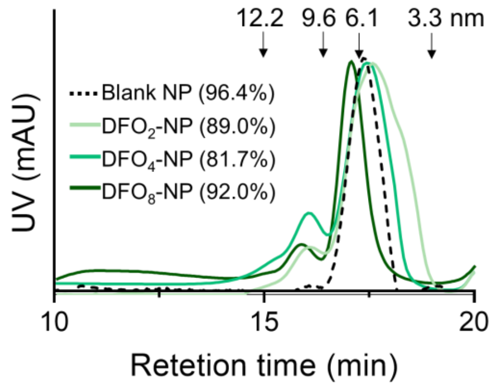

Supplementary Figure 7. GFC profiles of DFO-NPs to estimate hydrodynamic diameter (HD): Arrows and numbers indicate retention time maxima and corresponding HD of standard proteins, ribonuclease, 3.2 nm; ovalbumin, 6.1 nm; aldolase, 9.6 nm; ferritin, 12.2 nm.

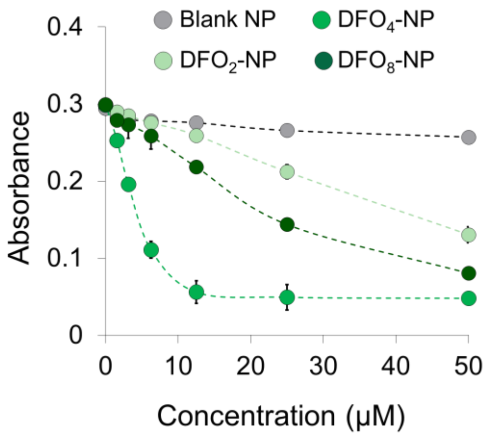

Supplementary Figure 8. UV absorbance curves of competitive iron binding assay as function of ferrozine concentration (0 to 50 μM). (n=3 mean ± SEM).

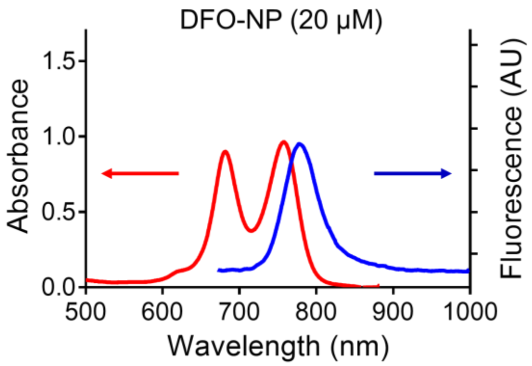

Supplementary Figure 9. Representative absorbance and fluorescence spectra of DFO-NPs in water.

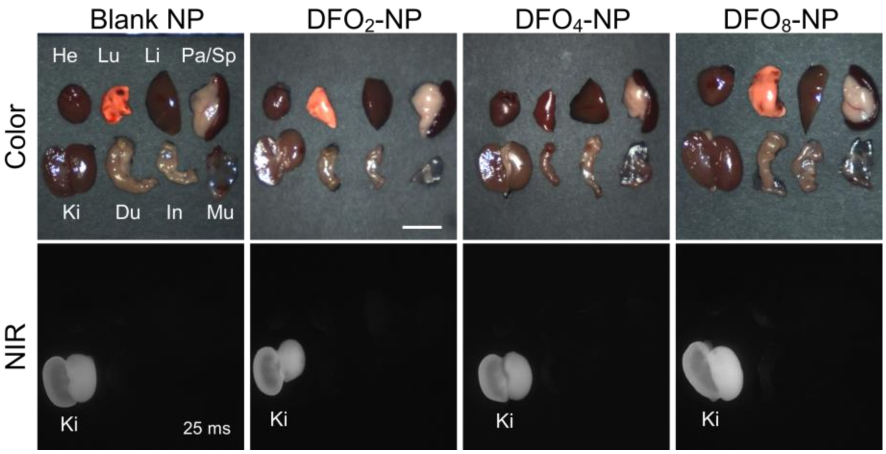

Supplementary Figure 10. Color and NIR fluorescence images of dissected organs of DFO-NPs (0.3 µmol kg^{-1}) treated mice at 4 h post-injection. He, heart; Lu, lung; Li, liver; Pa/Sp, pancreas/spleen; Ki, kidney; Du, duodenum; In, intestine; Mu, muscle. Scale bar: 1 cm.

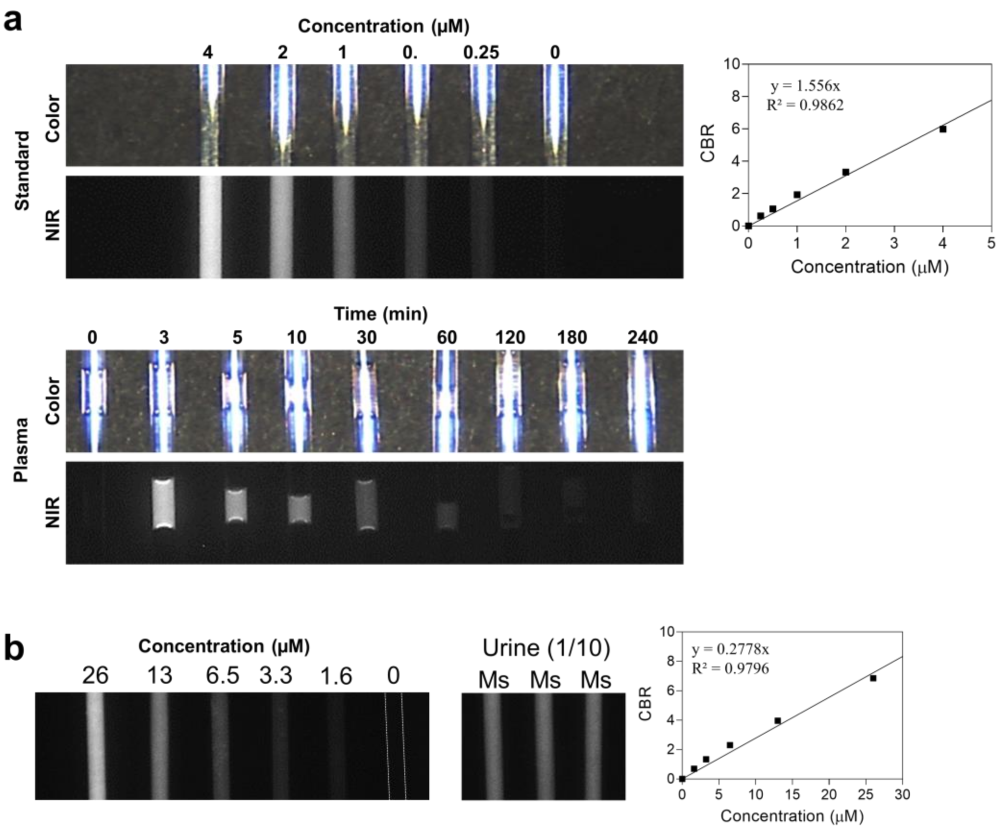

Supplementary Figure 11. Representative NIR images of (a) serum and (b) urine samples from DFO-NP treated mice and standard sample of DFO-NP with a serial dilution to quantify the amount of DFO-NPs in each sample.

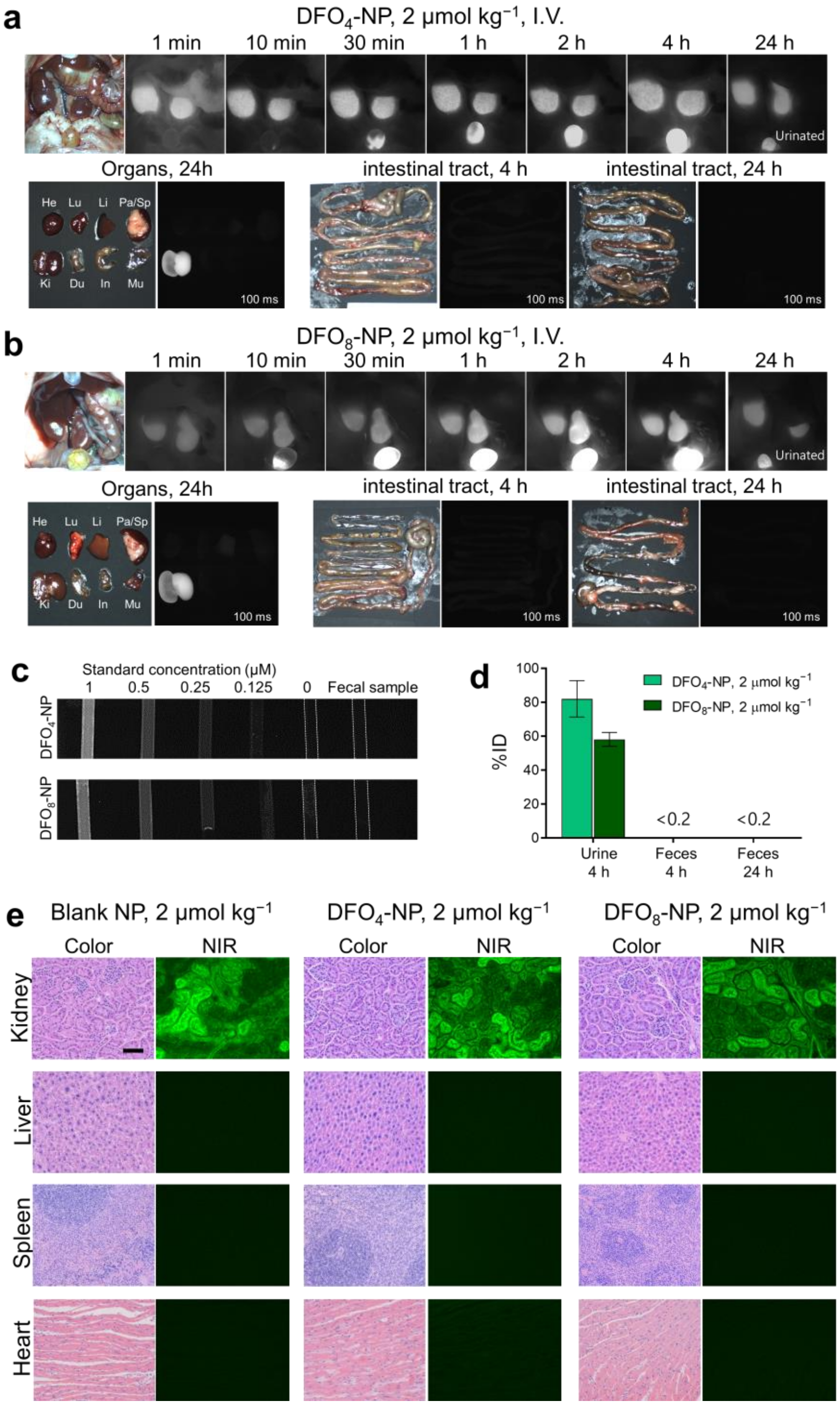

Supplementary Figure 12. **Evaluation of excretion route of DFO-NPs.** NIR images of abdominal cavity of CD-1 mice injected with (a) DFO4-NP (2 μmol kg^{-1}) and (b) DFO8-NP (2 μmol kg^{-1}). He, heart; Lu, lung; Li, liver; Pa/Sp, pancreas/spleen; Ki, kidney; Du, duodenum; In, intestine; Mu, muscle. (c, d) Quantitative analysis of fecal sample collected at 4 h and 24 h post injection. (n=3; mean ± SEM) (e) H&E staining and NIR fluorescence microscope images of kidney, liver, spleen, and heart. NIR fluorescence images indicate that DFO-NP can be excreted only through kidney filtration. Scale bar: 100 μm.

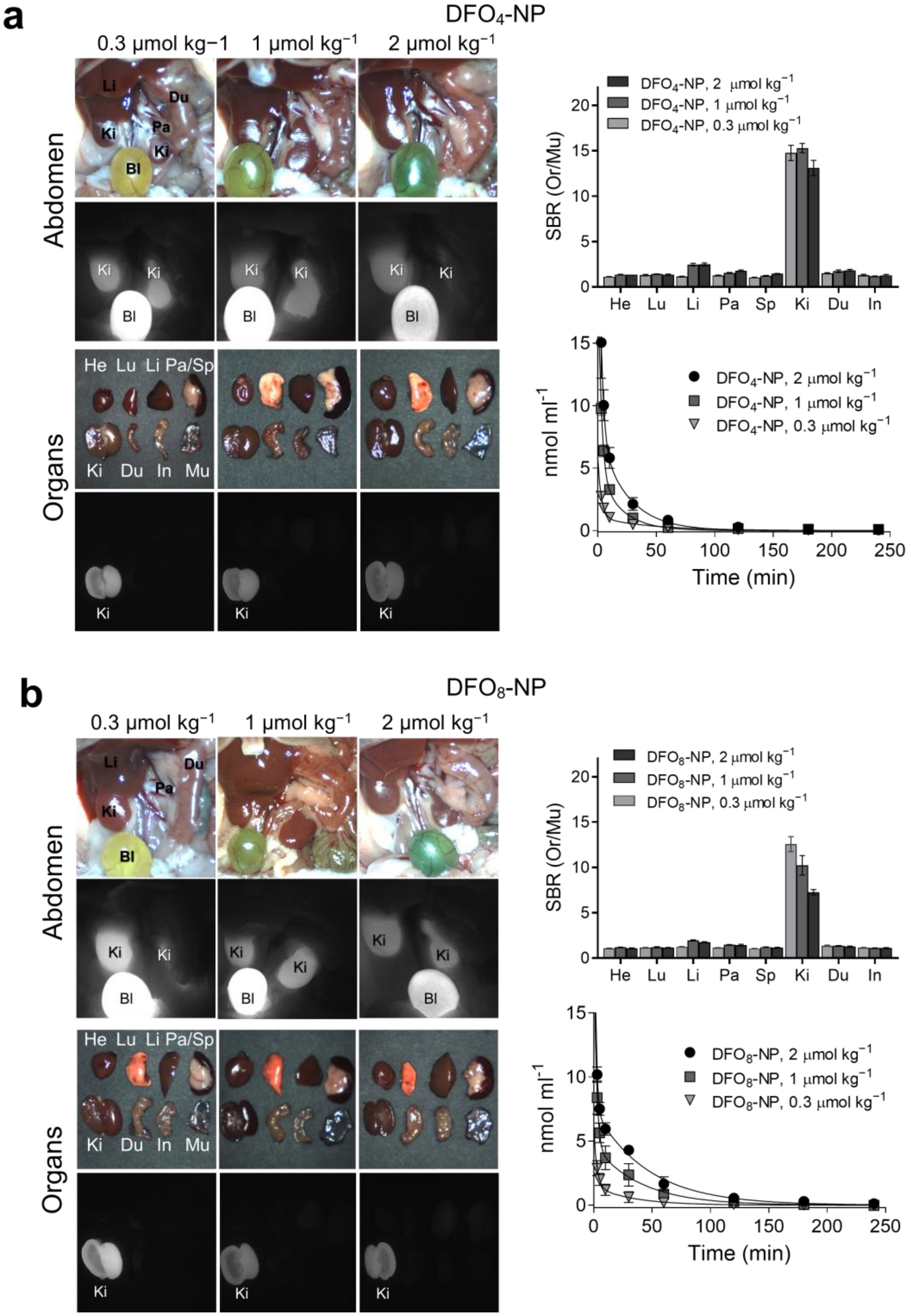

Supplementary Figure 13. Dose dependency on biodistribution PK of DFO-NPs: Color and NIR fluorescence images of mouse abdominal cavity and dissected organs of (a) DFO_4-NP and (b) DFO_8-NP. SBR of each organ against Mu. He, heart; Lu, lung; Li, liver; Pa/Sp, pancreas/spleen; Ki, kidney; Du, duodenum; In, intestine; Mu, muscle. Blood clearance curves of DFO-NPs (right, bottom). (n=3; mean ± SEM)

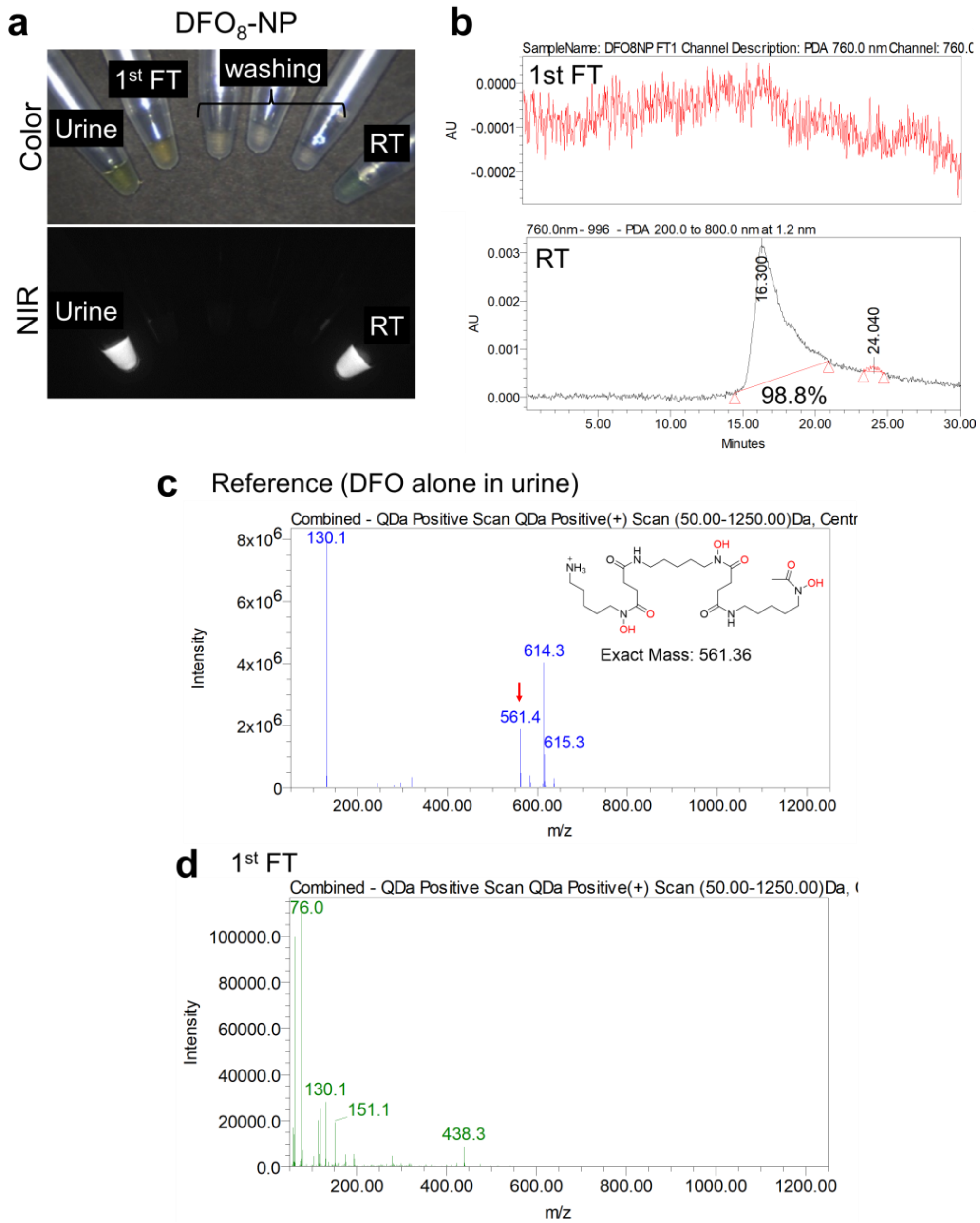

Supplementary Figure 14. Purification and analyses of collected urine samples: (a) Color and NIR image of collected urine from DFO$_8$-NP treated mice at 4 h post-injection, first flow-through, 1st FT; after washing with water 3 times, and retentive, RT after filtration with ultrafiltration unit (3,000 Da cutoff). (b) Retention time profile of DFO$_8$-NP by HPLC analysis. (c) Mass spectra of reference DFO sample in urine and (d) 1st FT of urine from DFO$_8$-NP treated mice.

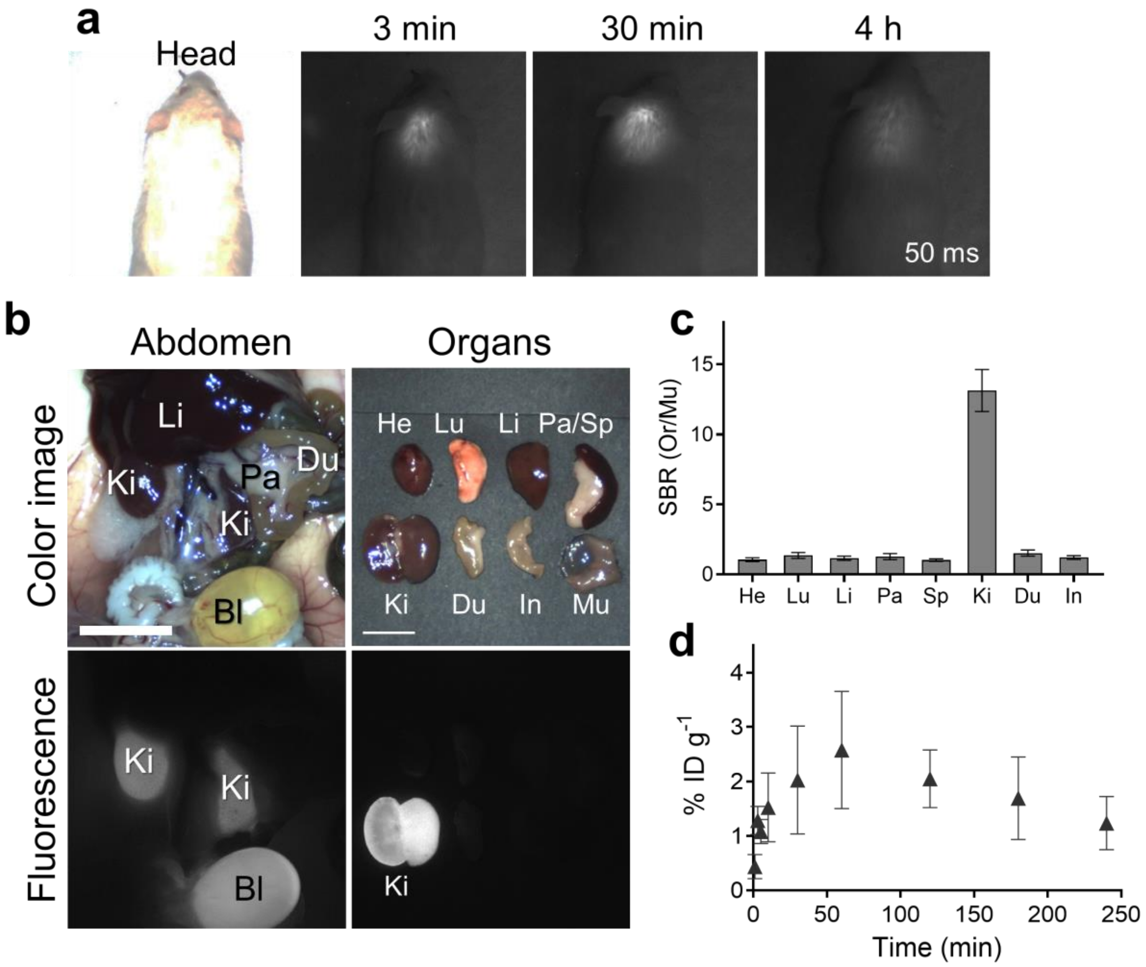

Supplementary Figure 15. PK and biodistribution of DFO_2-NP 4 h post-SC injection: (a) NIR image of a mouse in prone position after injection. (b) Intraoperative images of abdomen and dissected organs. Urinary excretion value was 10.72±3.81 %ID. (c) Biodistribution of DFO-NP in major resected organs. SBR of each organ against muscle (Mu) (n = 3 per group, mean ± SEM). He, heart; Lu, lung; Li, liver; Pa/Sp, pancreas/spleen; Ki, kidney; Du, duodenum; In, intestine; Mu, muscle. (d) Blood clearance curve of DFO-NP.

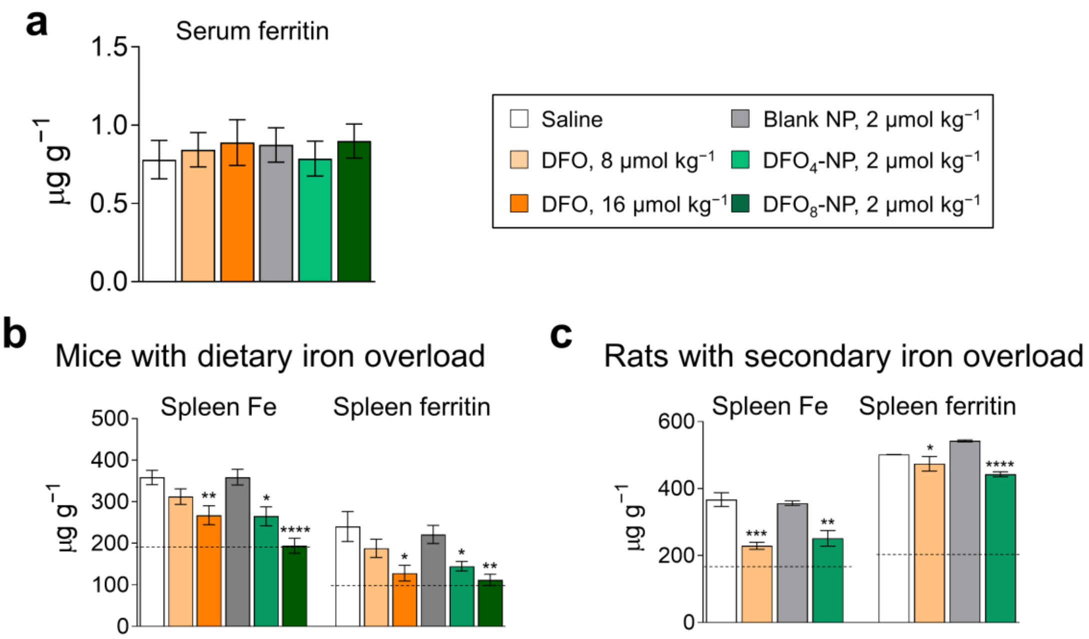

Supplementary Figure 16. Serum ferritin, spleen iron, and ferritin levels of iron overload mice and rats after DFO-NPs treatments. (a, b) CD-1 mice fed iron overload diet and (c) Belgrade (*b/b*) rats were subcutaneously injected with saline, native DFO and DFO-NPs daily for 5 d. (n = 3-8 per group, mean ± SEM.)

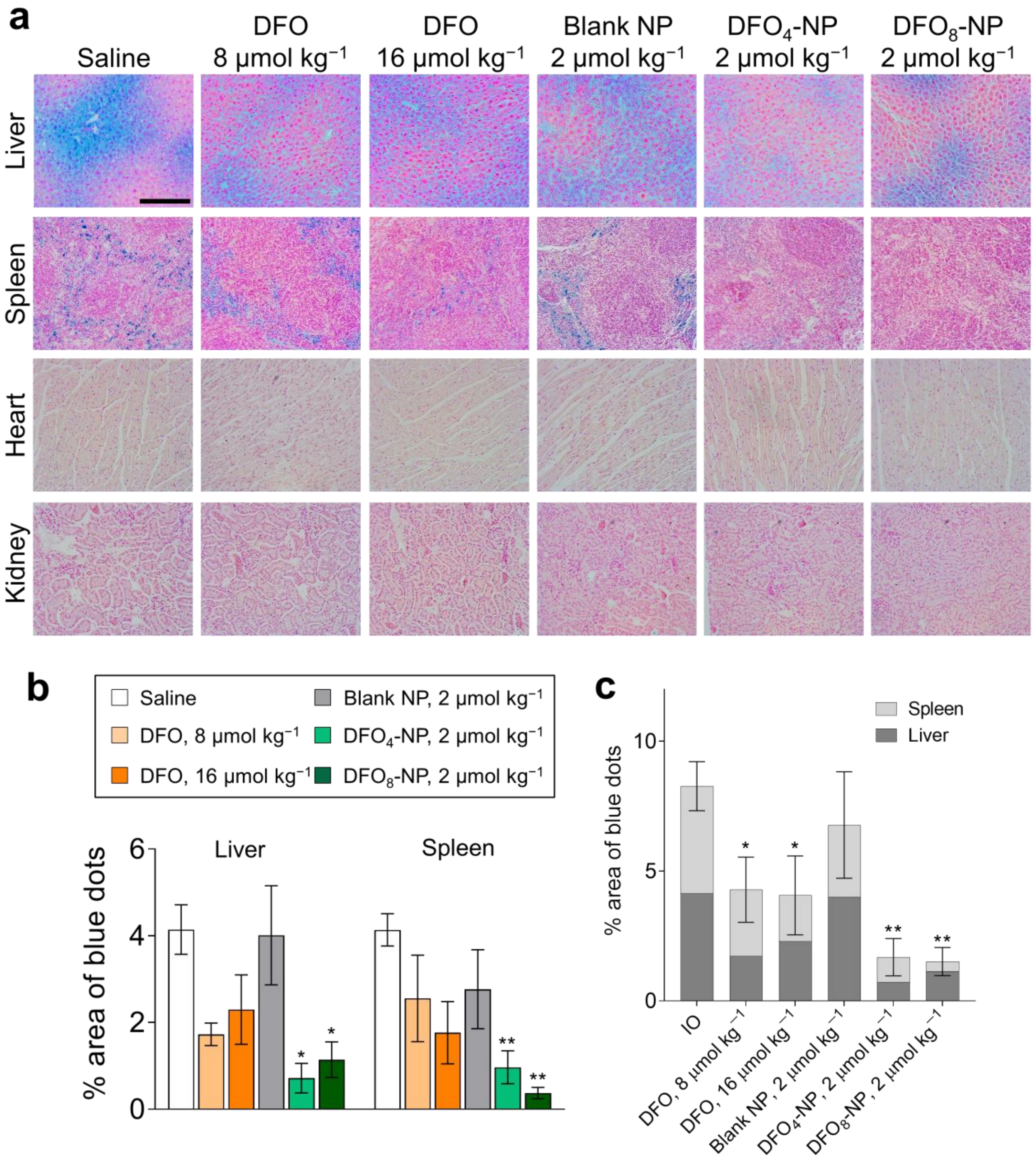

Supplementary Figure 17. **Prussian blue-stained sections of organs such as liver, spleen, heart, and kidney in iron overload mice following 5 days of daily treatments with saline, DFO, and DFO-NPs:** (a) Bright-filed images of Prussian blue-stained sections. Scale bar: 200 μm. The blue dots represent iron depositions in the tissues. (b) The calculated % area of blue dots in liver and spleen, and (c) cumulative value in liver and spleen. To obtain quantitative % area of blue dots, the area of the blue stained spots was calculated using ImageJ software. (n=5 per group; mean ± SEM)

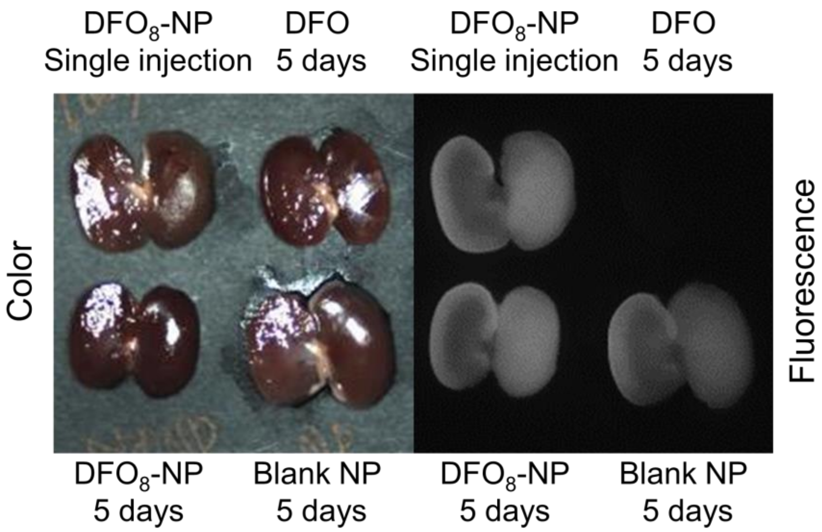

Supplementary Figure 18. Color and NIR images of kidney sections from CD-1 mice administered with native DFO alone (8 µmol kg^{-1}), blank NP (1 µmol kg^{-1}), DFO_8-NP (1 µmol kg^{-1} as EPL) daily SC injection for 5 days. Another CD-1 mouse was injected a single dose of DFO-NP (1 µmol kg^{-1}) on the 4th day.

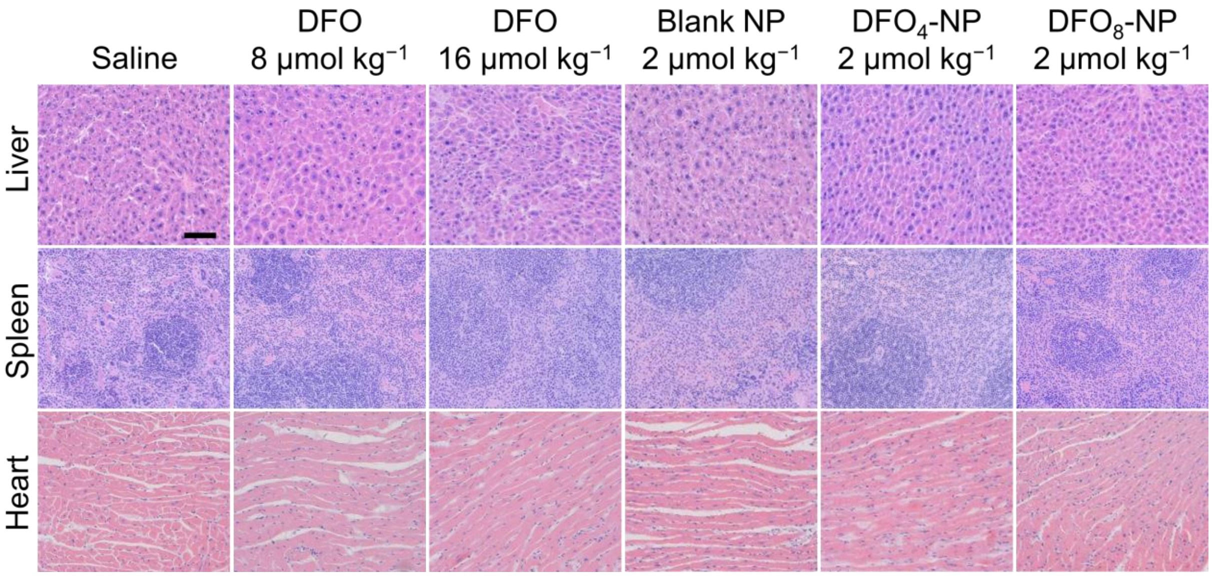

Supplementary Figure 19. **H&E-stained sections of liver, spleen, and heart** following 5 days of daily treatments with saline, DFO, and DFO-NPs as shown in Figure 3d. Scale bar: 100 µm.

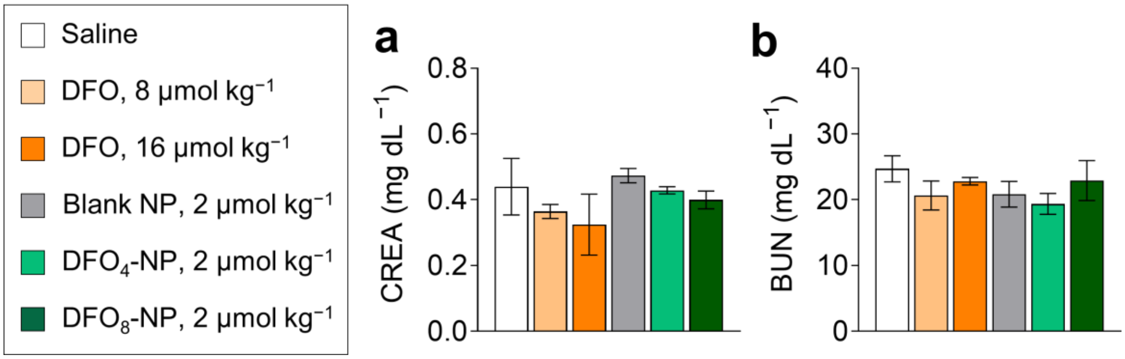

Supplementary Figure 20. Biochemical analysis of DFO-NPs in iron-overload animals. (a) Creatinine and (b) blood urea nitrogen (BUN) concentration from iron-loaded CD-1 mice treated with various concentrations of native DFO (8, 16, 80, and 160 μmol kg^{-1}) and DFO-NPs (2 μmol kg^{-1}). (n= 5; mean ± SEM)

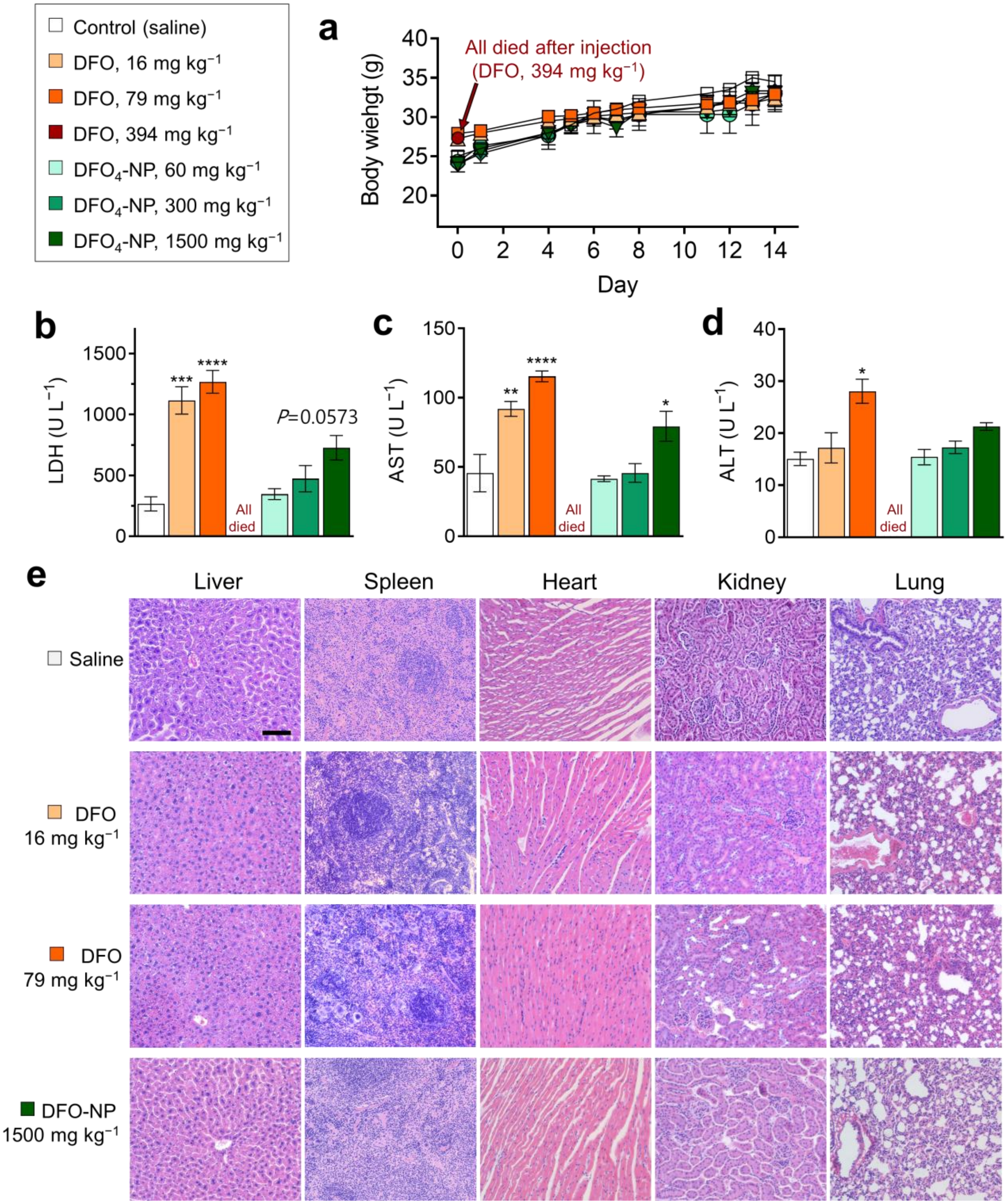

Supplementary Figure 21. Single dose acute toxicity test; biochemical analysis and tissue histology. (a) mouse body weight for 14 days after IV injection. (b) serum LDH, AST, and ALT levels in saline, DFO, and DFO_4-NP treatment groups. (n=4 to 5 per each group; mean ± SEM) (e) H&E staining of heart, liver, spleen, lung and kidney in each treatment group. Scale bar: 100 μm.

Supplementary References

1. Liu, Z., Qiao, J., Nagy, T. & Xiong, M.P. ROS-triggered degradable iron-chelating nanogels: Safely improving iron elimination in vivo. *J. Control. Release* **283**, 84-93 (2018).
2. Wang, Y., Liu, Z., Lin, T.M., Chanana, S. & Xiong, M.P. Nanogel-DFO conjugates as a model to investigate pharmacokinetics, biodistribution, and iron chelation in vivo. *Int. J. Pharm.* **538**, 79-86 (2018).
3. Yan, J. et al. Melanin nanoparticles as an endogenous agent for efficient iron overload therapy. *J. Mater. Chem. B* **4**, 7233-7240 (2016).
4. Hamilton, J.L. et al. In vivo efficacy, toxicity and biodistribution of ultra-long circulating desferrioxamine based polymeric iron chelator. *Biomaterials* **102**, 58-71 (2016).
5. Liu, Z., Lin, T.M., Purro, M. & Xiong, M.P. Enzymatically Biodegradable Polyrotaxane-Deferoxamine Conjugates for Iron Chelation. *ACS Appl. Mater. Interfaces* **8**, 25788-25797 (2016).
6. Liu, Z., Wang, Y., Purro, M. & Xiong, M.P. Oxidation-Induced Degradable Nanogels for Iron Chelation. *Sci. Rep.* **6**, 20923 (2016).
7. Imran ul-haq, M. et al. Design of Long Circulating Nontoxic Dendritic Polymers for the Removal of Iron in Vivo. *ACS Nano* **7**, 10704-10716 (2013).
8. Rossi, N.A. et al. In vitro chelating, cytotoxicity, and blood compatibility of degradable poly(ethylene glycol)-based macromolecular iron chelators. *Biomaterials* **30**, 638-648 (2009).
