# Supplementary material for: Renal clearable nanochelators for iron overload therapy
Source: Nat Commun. 2019 Nov 13;10:5134. doi: 10.1038/s41467-019-13143-z (PMC6853917; doi:10.1038/s41467-019-13143-z)
Supplement: Supplementary file 2 — Description of additional Supplementary Files [file 41467_2019_13143_MOESM2_ESM.docx]

**Description of Additional Supplementary Files**

File Name: Supplementary Movie 1

Description: Real time movie of mouse abdominal injected with DFO_4_-NP corresponding to Supplementary Figure 12a.

File Name: Supplementary Movie 2

Description: Real time movie of mouse abdominal injected with DFO_8_-NP corresponding to Supplementary Figure 12b.
