# Supplementary material for: Renal clearable nanochelators for iron overload therapy
Source: Nat Commun. 2019 Nov 13;10:5134. doi: 10.1038/s41467-019-13143-z (PMC6853917; doi:10.1038/s41467-019-13143-z)
Supplement: Supplementary file 5 — Reporting Summary [file 41467_2019_13143_MOESM5_ESM.pdf]

nature research

Corresponding author(s): Hak Soo Choi and Jonghan Kim

Last updated by author(s): Oct 17, 2019

Reporting Summary

Nature Research wishes to improve the reproducibility of the work that we publish. This form provides structure for consistency and transparency in reporting. For further information on Nature Research policies, see Authors & Referees and the Editorial Policy Checklist.

Statistics

For all statistical analyses, confirm that the following items are present in the figure legend, table legend, main text, or Methods section.

n/a	Confirmed	
☐	☒	The exact sample size (n) for each experimental group/condition, given as a discrete number and unit of measurement
☐	☒	A statement on whether measurements were taken from distinct samples or whether the same sample was measured repeatedly
☐	☒	The statistical test(s) used AND whether they are one- or two-sided<br>*Only common tests should be described solely by name; describe more complex techniques in the Methods section.*
☐	☒	A description of all covariates tested
☐	☒	A description of any assumptions or corrections, such as tests of normality and adjustment for multiple comparisons
☐	☒	A full description of the statistical parameters including central tendency (e.g. means) or other basic estimates (e.g. regression coefficient) AND variation (e.g. standard deviation) or associated estimates of uncertainty (e.g. confidence intervals)
☐	☒	For null hypothesis testing, the test statistic (e.g. F, t, r) with confidence intervals, effect sizes, degrees of freedom and P value noted<br>*Give P values as exact values whenever suitable.*
☒	☐	For Bayesian analysis, information on the choice of priors and Markov chain Monte Carlo settings
☒	☐	For hierarchical and complex designs, identification of the appropriate level for tests and full reporting of outcomes
☒	☐	Estimates of effect sizes (e.g. Cohen's d, Pearson's r), indicating how they were calculated

Our web collection on statistics for biologists contains articles on many of the points above.

Software and code

Policy information about availability of computer code

Data collection	NIR fluorescence images were obtained using the FLARE real-time intraoperative imaging system with customized FLARE software. An intraoperative fluorescence imaging system has been described in published paper (Ann. Surg. Oncol. 13, 1671–1681, 2006).
Data analysis	The NIR fluorescence intensities of region of interests were analyzed using customized FLARE software and ImageJ v1.51j8 (National Institutes of Health, Bethesda, MD, USA). The pharmacokinetic parameters were analyzed using SigmaPlot v12.3 (Systat Software Inc., San Jose, CA, USA). Graphpad Prism (version 8.12) was used for graphs and all other statistical analyses.

For manuscripts utilizing custom algorithms or software that are central to the research but not yet described in published literature, software must be made available to editors/reviewers. We strongly encourage code deposition in a community repository (e.g. GitHub). See the Nature Research guidelines for submitting code & software for further information.

Data

Policy information about availability of data

All manuscripts must include a data availability statement. This statement should provide the following information, where applicable:
- Accession codes, unique identifiers, or web links for publicly available datasets
- A list of figures that have associated raw data
- A description of any restrictions on data availability

The following statement is provided: The data that support the findings of this study are available from the authors on reasonable request, see author contributions for specific data sets.

Field-specific reporting

Please select the one below that is the best fit for your research. If you are not sure, read the appropriate sections before making your selection.

☒ Life sciences ☐ Behavioural & social sciences ☐ Ecological, evolutionary & environmental sciences

For a reference copy of the document with all sections, see nature.com/documents/nr-reporting-summary-flat.pdf

Life sciences study design

All studies must disclose on these points even when the disclosure is negative.

Sample size	Sample size calculations were not performed. Sample size was determined to be adequate based on the magnitude and consistency of measurable differences between groups. 3-8 mice were used per each group for imaging, biodistribution, and pharmacokinetic studies. The number of mice/rats was dependent on the yield of usable mice/rats.
Data exclusions	No data were excluded.
Replication	Replicate experiments were successful.
Randomization	Animals were assigned randomly to experimental and control groups, and within animal controls were performed wherever possible.
Blinding	For histological evaluation, the sample information was blinded.

Reporting for specific materials, systems and methods

We require information from authors about some types of materials, experimental systems and methods used in many studies. Here, indicate whether each material, system or method listed is relevant to your study. If you are not sure if a list item applies to your research, read the appropriate section before selecting a response.

Materials & experimental systems

n/a	Involved in the study	
☒	☐	Antibodies
☒	☐	Eukaryotic cell lines
☒	☐	Palaeontology
☐	☒	Animals and other organisms
☒	☐	Human research participants
☒	☐	Clinical data

Methods

n/a	Involved in the study	
☒	☐	ChIP-seq
☒	☐	Flow cytometry
☒	☐	MRI-based neuroimaging

Animals and other organisms

Policy information about studies involving animals; ARRIVE guidelines recommended for reporting animal research

Laboratory animals	Details on the mice used in this study have been included in the methods section of the manuscript. CD-1 mice (male; 30-35 g; Charles River Laboratories) were used for biodistribution and pharmacokinetic studies. CD-1 mice feeding with high iron-diet for a week and Heterozygous (+/b) and homozygous (b/b) Belgrade rats (Fischer F344 background) were used for iron overload rodent models.
Wild animals	The study did not involve wild animals.
Field-collected samples	The study did not involve samples collected from the field.
Ethics oversight	Animals were housed in an AAALAC-certified facility and studied under the supervision of MGH IACUC in accordance with the approved institutional protocol (#2016N000136) and Northeastern University IACUC in accordance with the approved institutional protocols (16-0305R/18-0310R).

Note that full information on the approval of the study protocol must also be provided in the manuscript.
